# The intricacies of *Acinetobacter baumannii*: a multifaceted comprehensive review of a multidrug-resistant pathogen and its clinical significance and implications

**DOI:** 10.3389/fmicb.2025.1565965

**Published:** 2025-05-12

**Authors:** Amani Yehya, Zeinab Ezzeddine, Mohamed Chakkour, Zahraa Dhaini, Miriama S. Bou Saba, Anthony S. Bou Saba, Lea Nohra, Nagham B. Nassar, Mahdi Yassine, Hisham F. Bahmad, Ghassan Ghssein

**Affiliations:** ^1^Department of Anatomy, Cell Biology, and Physiological Sciences, Faculty of Medicine, American University of Beirut, Beirut, Lebanon; ^2^High Council for Scientific Research and Publication (HCSRP), Islamic University of Lebanon (IUL), Khalde, Lebanon; ^3^Department of Biological Sciences, Wayne State University, Detroit, MI, United States; ^4^Faculty of Medical Sciences, Lebanese University, Beirut, Lebanon; ^5^Department of Pathology and Laboratory Medicine, University of Miami Miller School of Medicine, Miami, FL, United States

**Keywords:** *Acinetobacter baumannii*, multidrug-resistant, nosocomial, pathogen, review

## Abstract

*Acinetobacter baumannii*, a highly adaptive and formidable nosocomial pathogen, has emerged as a symbol of modern medicine's struggle against multidrug resistance (MDR). As a Gram-negative dweller in moist hospital environments, *A. baumannii* has proven its ability to colonize the most vulnerable—critically ill patients—leaving behind a trail of infections highlighted by high morbidity and mortality and rendering nearly all antibiotics ineffective. This literature review aims to provide an in-depth, comprehensive overview of microbiological features, virulence factors, clinical manifestations, epidemiology, and antibiotic resistance mechanisms of *A. baumannii*. It also highlights the different diagnostic approaches, possible treatment strategies, and infection control, as well as the profound public health burden this pathogen imposes. The genus *Acinetobacter* has undergone a pivotal taxonomic journey and categorization. In addition, the intricate virulence mechanisms and factors of *A. baumannii*, including but not limited to outer membrane components and nutrient acquisition systems, have contributed to its pathogenicity and severe clinical manifestations ranging from respiratory tract infections and meningitis to urinary tract infections, skin infections, and bloodstream infections. This review also describes the epidemiological trend of *A. baumannii* established by its global prevalence and distribution, risk factors, hospital-acquired vs. community-acquired infections, and its geographical variations. In terms of antibiotic resistance, this pathogen has demonstrated resilience to a wide range of first-line and last-resort antibiotics due to its different evasion mechanisms. The current diagnostic approaches, treatment strategies, and infection control measures are further analyzed in detail, underscoring the need for prompt and precise identification of *A. baumannii* to guide appropriate therapy and reinforce the optimal approaches to limit its transmission and control outbreaks. Finally, the review addresses the substantial public health implications, reflecting on the hindrance that *A. baumannii* brings to healthcare systems, and the urgent need for global surveillance, effective infection control protocols, innovative research, and therapeutic approaches to mitigate its global threat.

## 1 Introduction

In the labyrinth of microbial biology and pathology, *Acinetobacter baumannii* stands out as a puzzling challenge for modern healthcare systems. Dating back to the early 20th century, *A. baumannii* is recognized as a strictly aerobic, Gram-negative, non-fermenting, non-fastidious, non-motile, catalase-positive, and oxidase-negative bacterium (Almasaudi, 2018; Jung and Park, 2015). This organism is highly prevalent in moist hospital environments, where it is notorious for its propensity to colonize the skin of hospitalized patients, leading to nosocomial infections that account for over 20% of all hospital-acquired infections (HAIs) in intensive care units (ICUs; Ayobami et al., 2019).

In recent years, *A. baumannii* has been classified as one of the World Health Organizations' (WHO) critical priority pathogens due to its remarkable ability to develop resistance to multiple last-resort antibiotics. Even during the COVID-19 pandemic, the challenges posed by *A. baumannii* infections, particularly those resistant to carbapenems, intensified the burden on healthcare systems that were already overwhelmed by the virus outbreak (Kyriakidis et al., 2021; Thoma et al., 2022). Thus, it is imperative to have a comprehensive understanding of *A. baumannii*'s epidemiology, virulence factors, clinical manifestations, and antibiotic resistance mechanisms.

A key factor contributing to the persistence and multidrug resistance (MDR) of *A. baumannii* is its genetic plasticity. This adaptability enables rapid genetic mutations and the integration of foreign genetic elements, facilitating swift adaptation to hostile hospital environments. Such genetic flexibility enhances its antibiotic resistance, virulence, and environmental resilience, making it particularly challenging to eradicate from healthcare settings (Kyriakidis et al., 2021; Scoffone et al., 2025).

Despite the extensive research on *A. baumannii*, many studies have taken fragmented approaches, focusing on either epidemiological trend, resistance mechanisms, or clinical manifestations in isolation. This review aims to provide a comprehensive and integrative analysis of *A. baumannii*, covering its epidemiology, genetic evolution, virulence factors, diagnostic approaches, therapeutic strategies, and infection control measures. By referring to recent findings, this review seeks to bridge existing knowledge gaps and offer a holistic perspective on the burden of *A. baumannii* on public health.

## 2 Microbiological features

The genus *Acinetobacter* has undergone an intriguing taxonomic evolution, with early challenges in classification due to a lack of distinctive morphological and biochemical features. In 1970, advancements in transformation and nutritional studies enabled the formal definition and classification of the family *Neisseriaceae* (Henriksen, 1976). Initially, *Acinetobacter* was classified based on a set of distinctive traits: lack of color, non-motility, inability to reduce nitrate, and non-fermentative characteristics (Cowan, 1938). Brisou and Prevot later characterized *Acinetobacter* as colorless, non-motile, saprophytic Gram-negative *bacilli*, irrespective of their oxidase activity (Brisou and Prevot, 1954). As research progressed, advancements in the medical field led to classifications based on the bacterium's oxidase activity, distinguishing between oxidase-positive bacteria, referred to as *Moraxella*, and oxidase-negative bacteria, referred to as *Acinetobacter* (Gerner-Smidt et al., 1991). By 1971, a clearer understanding of the genus began to emerge, although species were still not well established (Juni, 1972). Prior to the mid-1980s, based on available references, *Acinetobacter calcoaceticus* (*A. calcoaceticus*) was the sole recognized species, divided into two subspecies: var. *anitratus* and var. *lwoffii*, differentiated by their ability to ferment glucose (Juni, 1972; Bauman et al., 1978). With advancements in bacterial characterization methods, more species have been identified, and the genus classification has been expanded (Shelenkov et al., 2023).

Today, over 50 species of *Acinetobacter* have been identified, the majority of which are non-pathogenic. Among these, the *A. calcoaceticus*-*baumannii* (Acb) complex stands out as a phylogenetically pathogenic species, clinically prevalent in human infections (Touchon et al., 2014). This complex includes five pathogenic species: *A. baumannii, Acinetobacter nosocomialis, Acinetobacter pittii, Acinetobacter seifertii*, and *Acinetobacter dijkshoorniae*. along with non-pathogenic species *A. calcoaceticus* (Bouvet and Grimont, 1986; Nemec et al., 2011, 2015; Cosgaya et al., 2016). The taxonomic complexity within this complex has substantial clinical implications, especially in terms of antimicrobial resistance (AMR) and clinical outcomes. *A. baumannii*, the most clinically significant species within this complex, is notorious for its MDR, including resistance to last-resort treatments in severe infections (Holmström et al., 2022). The varying AMR profiles across the Acb complex underscore the importance of precise species identification in clinical microbiology. Accurate species-level identification, facilitated by advanced molecular techniques, is crucial for guiding clinicians in selecting the most appropriate therapy. Misidentifying species within the Acb complex can lead to inappropriate treatment, resulting in treatment failures and the further spread of resistant strains (Vijayakumar et al., 2019; Kim and Mun, 2025).

Molecular typing methods, particularly multilocus sequence typing (MLST), have become crucial for understanding the epidemiology of *A. baumannii*. MLST was proposed in the 20th century as the gold standard technology for *Neisseria meningitidis*, although this title later shifted to *A. baumannii*. MLST involves sequencing 400–500 bp internal fragments of typically seven housekeeping genes (*gltA, gyrB, gdhB, recA, cpn60, gpi*, and *rpoD*), providing a standardized method for characterizing bacterial isolates. Each species possesses characteristic alleles for each gene, and different combinations of alleles across the seven genes define a sequence type (ST; Bartual et al., 2005; Maiden et al., 2013). The MLST database, PubMLST.org, serves as a global repository for these sequences, offering valuable information for tracking the spread of resistant strains (Jolley et al., 2018).

**Figure 1:**
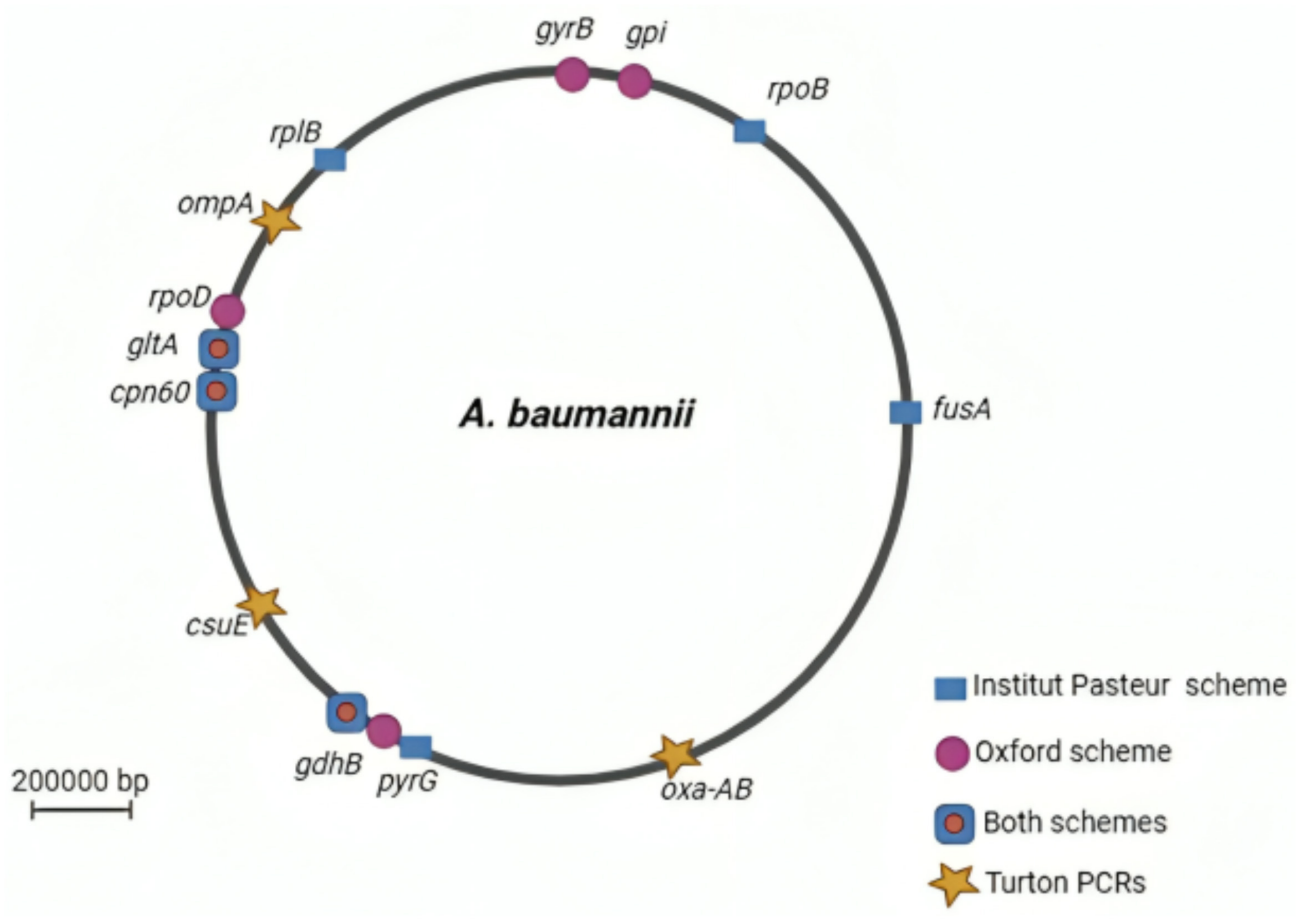
A circular map of the *Acinetobacter baumannii* chromosome depicting the locations of genes used in the Oxford and Institut Pasteur MLST methods. Stars (Turton PCRs) denote genes found by trilocus typing by PCR. Created in BioRender. ezz, z. (2025) https://BioRender.com/a0dpfdb.

Two MLST schemes, namely, Pasteur and Oxford schemes, were proposed for *A. baumannii* (Diancourt et al., 2010; Bartual et al., 2005). The Pasteur scheme includes *cpn60, fusA, gltA, pyrG, recA, rplB*, and *rpoB* genes, while the Oxford scheme utilizes *gltA, gyrB, gdhB, recA, cpn60, gpi*, and *rpoD*. Although three genes are common to both schemes, the Oxford scheme is more discriminant for closely related isolates, despite facing challenges such as *gdhB* gene paralogy, recombinations, and technical artifacts (Figure 1). In contrast, the Pasteur scheme appears to be less affected by homologous recombination and is better suited for accurate classification within clonal groups. As on 25 March 2023, the PubMLST database contained 2,262 sequence types for Pasteur profiles and 2,850 for Oxford profiles (Gaiarsa et al., 2019; Hamidian et al., 2017). However, the Oxford scheme remains less discriminative than the pulsed-field gel electrophoresis (PFGE) test, despite its reported significant role in resolving various outbreaks, including identifying the *gpi* and *gyrB* genes. Moreover, analysis using the Oxford scheme and PFGE of *A. baumannii* from different outbreaks and countries revealed sequence types with the same subdivision. In contrast, the Pasteur scheme revealed no variations between outbreaks (Tomaschek et al., 2016).

Capsule synthesis loci (K, or KL loci) and lipopolysaccharide outer core loci (OCL) can significantly contribute to isolating typing and classification purposes (Wyres et al., 2020; Arbatsky et al., 2018). The capsule polysaccharide (CPS) gene cluster comprises approximately 30 genes, whereas the OC locus comprises only five genes. Each distinct gene cluster found between the flanking gene clusters is assigned a unique number, designating the locus type. CPS is a valuable epidemiological marker due to its role in bacterial virulence and susceptibility to phages. Similarly, the OCLs exhibit variation in *A. baumannii*. Like MLST, gene sequences and matching KL and OCL types are stored in a public database (Wyres et al., 2020; Cahill et al., 2022). Additionally, typing methods can be employed using dedicated software online or offline (Lam et al., 2022). This categorization suggests further epidemiological value, especially when combined with other systems like MLST, as there are only a few KL and OCL types for *A. baumannii* (237 and 22, respectively, as of March 20, 2023), compared to the number of MLST profiles (Tyumentseva et al., 2021).

## 3 Virulence factors and pathogenesis

### 3.1 Pathogenesis of *A. Baumannii*

#### 3.1.1 Primary stage of infection

When discussing the methods and extent of pathogenicity of *A. baumannii*, emphasis should be placed on establishing contact through adherence or adhesion of the bacterium to the host's surface. Contact is thus a primary step for pathogenicity, preceding the activation of *A. baumannii*'s virulence factors that facilitate substantial colonization (Falagas and Rafailidis, 2007).

Although considerable time may pass before the bacterium contacts its host, time does not pose a threat to *A. baumannii*, as this organism can survive for up to 5 months on inanimate surfaces (Kramer et al., 2006) and up to 9 days on hospital bed rails after the discharge of infected patients (Fournier and Richet, 2006). These extended survival times may explain the high rates of nosocomial cross-contamination (Pendleton et al., 2013), compensating for the bacterium's reduced capacity to adhere to cells, including mucosal cells, compared to other bacteria, such as *Pseudomonas aeruginosa, Neisseria. meningitides, Campylobacter, Yersinia enterocolitica*, and *Helicobacter pylori* (Asif et al., 2018).

Additionally, due to its hydrophobic properties, *A. baumannii* can increase the likelihood of establishing infection by attaching to foreign materials, such as plastics and catheters. Hydrophobicity refers to the bacterium's ability to adhere to surfaces that repel water. Research has shown that A. baumannii strains isolated from hospitalized patients tend to exhibit greater surface hydrophobicity compared to normal skin flora, which may enhance their ability to adhere to medical devices and cause infections (Peleg et al., 2008). A significant proportion of these organisms in hospitalized patients remain colonized rather than causing active infection. This suggests that *A. baumannii* strains in colonized patients have less pronounced hydrophobicity, making them less likely to adhere to host tissues or medical devices compared to strains in infected individuals (Fournier and Richet, 2006).

Moreover, once contact has been established, *A. baumannii* employs a “persist and resist” strategy, transitioning its growth mode from free-swimming to surface-oriented (Mea et al., 2021).

#### 3.1.2 Motility

In the effort for *A. baumannii* to establish efficient cell-to-cell adhesion and cause disease, the bacterium must be in a free-moving state, employing various techniques to achieve motility, which is essential for targeting (Mea et al., 2021; Josenhans and Suerbaum, 2002). Indeed, a study by Skiebe et al. demonstrated that majority of the *A. baumannii* organisms exhibit some degree of motility on wet surfaces, which may explain the extensive colonization of the bacterium on hospital equipment (Skiebe et al., 2012).

*A. baumannii* and other *Acinetobacter* species can utilize a mode of motility called twitching movement, which involves using type-IV pili (TFP) to cause retraction, thus propelling the bacterial cell forward (Jarrell and McBride, 2008). Moreover, some *A. baumannii* species have shown both surface-associated movement (swarming motility), enabling movement on semi-solid surfaces (Barker and Maxted, 1975), and twitching motility (Skiebe et al., 2012; Eijkelkamp et al., 2011). Additionally, the swarming motility described by Barker and Maxted in 1975 (Barker and Maxted, 1975) is dependent on the *pilT* gene, with the loss of this gene resulting in a 54% decrease in organism motility but not abolishing it (Clemmer et al., 2011; Wilharm et al., 2013). Furthermore, among the genes known to constitute pili assembly, only *pilR, pilS, pilT*, and *pilU* are involved in twitching (Clemmer et al., 2011).

Furthermore, the extent of motility differs between isolates, where a study by Dahdouh et al. showed an increase in motility in Lebanese isolates compared to Spanish isolates (Dahdouh et al., 2018). Therefore, not all isolates can have the perfect conditions to thrive, and motility can be decreased according to the presence of iron-limiting conditions (Eijkelkamp et al., 2011), mutation transposon insertions in *dat* and *ddc* genes (Skiebe et al., 2012), amino acid substitutions in position 520 or 540 of the RpoB protein (Pérez-Varela et al., 2017), light, salinity, LPS, and other extracellular conditions (Mea et al., 2021; Josenhans and Suerbaum, 2002; Corral et al., 2020; Talà et al., 2019).

Collectively, this highlights a promising avenue for researching innovative therapeutic approaches to abolish motility.

#### 3.1.3 Features related to A. baumannii's surface

Once *A. baumannii* identifies a suitable target, overcoming the repulsive forces between its surface and the target's surface becomes imperative (Mea et al., 2021). This is achieved through the establishment of initially weak and reversible hydrophobic interactions, which become long-lasting over time (Rosenberg et al., 2013).

Therefore, a crucial feature observed in *A. baumannii* is cell surface hydrophobicity, enabling the bacterium to access hydrocarbon sources (Krasowska and Sigler, 2014) and adapt to new environments (Mea et al., 2021). Furthermore, numerous studies emphasize the role of cell surface hydrophobicity in biofilm formation on medical equipment, thereby enhancing adherence ability (Pour et al., 2011).

A proposed characteristic of *A. baumannii*'s surface is its ability to modulate hydrophobic and hydrophilic interactions according to its requirements, effectively utilizing the repulsive and attractive forces necessary for subsequent adhesion to and invasion of the host (Khelissa et al., 2017; Vij et al., 2020).

#### 3.1.4 The adherence and initiation of an infection

A critical transcriptional change must occur for *A. baumannii* to tightly adhere to its new habitat, wherein the bacterium downregulates motility-associated genes and upregulates biofilm-forming genes (Rumbo-Feal et al., 2013). This transition is essential for shifting from a free-moving state to a surface-colonizing state (Mea et al., 2021). Several studies have emerged to evaluate the role of pili in the adherence and colonization profile of *A. baumannii*, prompted by the widespread conservation of pili in all *A. baumannii* isolates (Moriel et al., 2013; Tomaras et al., 2003).

For this reason, the *csuA/B* gene has been extensively studied, as it encodes for the CsuA/BABCDE-mediated pilus (Pakharukova et al., 2017), which is not as crucial for the direct attachment of *A. baumannii* to its target cells, but rather highly essential for biofilm formation on abiotic surfaces (de Breij et al., 2010; Lee et al., 2006).

A biofilm-associated protein (Bap) has been identified in *A. baumannii*, contributing to biofilm production and differentiation into maturity phases (Brossard and Campagnari, 2012; Loehfelm et al., 2008). There is heterogeneity in this protein among *A. baumannii* strains, which may account for variations in biofilm formation effectiveness among these strains (De Gregorio et al., 2015). Studies have extensively investigated the role of this gene, revealing that Bap increases cell surface hydrophobicity, thereby enhancing attachment and adherence abilities (Brossard and Campagnari, 2012). Other proteins known as Bap-like proteins (BLP1 and BLP2) have also been isolated from different *A. baumannii* species and are believed to regulate adherence by reducing its effectiveness when co-expressed with the Bap protein (De Gregorio et al., 2015).

In addition, other factors contribute to the adhesion profile of this organism, such as the β-lactamase gene, which participates in biofilm formation (Lee et al., 2008; Bardbari et al., 2017; Yang et al., 2019), and the autotransporter adhesion (ATA) protein, which mediates bacterial adhesion to extracellular matrix (ECM) proteins (Bentancor et al., 2012a; Weidensdorfer et al., 2015).

It is noteworthy that the ATA protein elicits a severe and intensive immune response, highlighting its significant role in the infection process (Weidensdorfer et al., 2015; Thibau et al., 2020). Indeed, infection with *A. baumannii* can induce both known types of immune responses: the extensively studied innate immune response and the adaptive immune response, which requires further investigation for a comprehensive understanding (Morris et al., 2019).

During infection initiation, *A. baumannii* affects various cells of the innate immune system, including neutrophils, macrophages, natural killer (NK) cells, mast cells, and dendritic cells. In the early phase of infection, neutrophils play a crucial role by rapidly phagocytosing bacterial cells, employing defensive mechanisms that can take as little as 20 s to activate (Morris et al., 2019; Nordenfelt and Tapper, 2011). These defense strategies involve immunoglobulin G (IgG)-mediated opsonization, toll-like receptor (TLR) activation, and complement-mediated binding to neutrophil receptors (Nordenfelt and Tapper, 2011). Despite neutrophils' efforts within the first 4–24 h of infection (Bruhn et al., 2015; García-Patiño et al., 2017), *A. baumannii* can induce neutrophil scattering by binding to neutrophil surfaces in an interleukin-8 (IL-8)-dependent manner (Kamoshida et al., 2016).

Macrophages play a complex role in defense against *A. baumannii*, exhibiting a slower phagocytic response compared to neutrophils, which may take a minimum of 10 min and occurs at a slower rate (Morris et al., 2019; Qiu et al., 2012; Lázaro-Díez et al., 2017). Despite this, their contribution in the early stages of infection cannot be discounted, as they also play a role in recruiting neutrophils. In an *in vivo* study utilizing a mouse model of intranasal infection, early depletion of macrophages during infection has been shown to increase disease severity (Qiu et al., 2012).

Furthermore, *A. baumannii* has developed mechanisms to evade macrophage immunity, including inducing autophagocytosis of macrophages. This evasion strategy is facilitated by the OmpA protein, which acts on several signaling pathways, including the mitogen-activated protein kinase (MAPK)/c-Jun N-terminal kinase (JNK) signaling pathway (An et al., 2019) and the mammalian target of rapamycin (mTOR) pathway (Zhao et al., 2021).

In addition to phagocytic cells, NK cells also play a crucial role, although further investigation is needed to understand their contribution fully. In mice with *A. baumannii* pneumonia, depletion of NK cells has been shown to increase mortality rates, underscoring their importance (Tsuchiya et al., 2012). Mast cells, in contrast, secrete tissue necrosis factor-α and interleukin-8, which promote the function of neutrophils (Kikuchi-Ueda et al., 2017).

It is suggested that *A. baumannii* pathogenicity is highly dependent on this mechanism, as the pathogen may modulate OmpA expression to evade the T cell response, particularly T helper 1 cells, and hinder progression toward a more specific adaptive immunity characterized by lymphocyte proliferation and maturation (Lee J. S. et al., 2010).

### 3.2 Virulence factors of *A. Baumannii*

**Figure 2:**
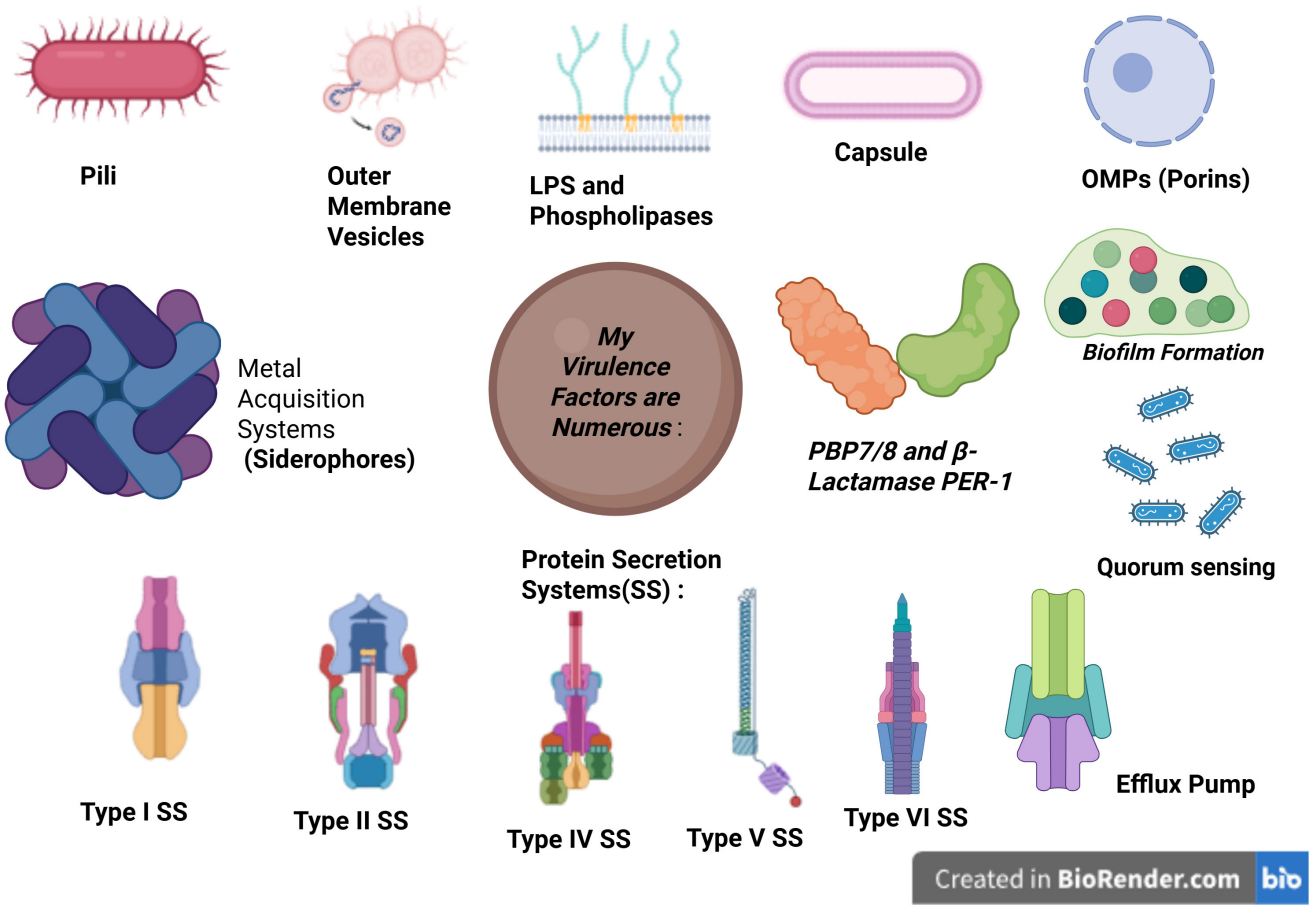
Schematic representation of the major virulence factors of *Acinetobacter baumannii*. Created in BioRender. b, a. (2025) https://BioRender.com/nflrr8d. OMPs, outer membrane vesicles.

*A. baumannii* is equipped with various virulent factors that enable it to survive in hostile environments and evade the host's immune defenses. These factors contribute to its pathogenicity, making infections difficult to treat and control (Figure 2).

#### 3.2.1 Outer membrane components

##### 3.2.1.1 Outer membrane proteins (porins)

Outer membrane proteins (OMPs), also known as porins, are found on the surface of the outer membrane of *A. baumannii* (Lee J. S. et al., 2010). These proteins facilitate the transport of solutes across the bacterium's membranes, with the outer membrane enabling for more diffuse transport compared to the inner cytoplasmic membrane. OMPs typically constitute approximately half of the outer membrane (Mea et al., 2021), and are significant virulence factors of *A. baumannii*, contributing to various aspects of its pathogenicity, including antibiotic resistance.

The first identified and majority of the well-known porin in *A. baumannii* is OmpA (previously called Omp 38), which is a β-barrel porin with a 2-nm pore diameter (Sugawara and Nikaido, 2012). OmpA exhibits low heterogeneity (Kim et al., 2016) and lower permeability compared to similarly sized porins in other bacteria, such as *Escherichia coli*, highlighting its significance in antimicrobial resistance (Sugawara and Nikaido, 2012; Kwon et al., 2019).

Furthermore, OmpA enables *A. baumannii* to attach to epithelial cells of the respiratory tract by binding to and interacting with fibronectin on the surface of lung cells (Smani et al., 2012a,b). Moreover, OmpA constitutes a significant portion of outer membrane vesicles (OMVs), which are another virulence factor in this bacterium, and can induce premature apoptosis of dendritic cells (Lee J. S. et al., 2010).

Additionally, OmpA can induce cytotoxicity in cells by binding to eukaryotic cell surface death receptors (Al Atrouni et al., 2016a). Upon internalization by healthy cells, OmpA translocates to either the mitochondria or the nucleus (Choi et al., 2005; Rumbo et al., 2014), where it activates Bcl-2 family proteins, leading to pro-apoptotic signals responsible for the release of cytochrome C and apoptosis-inducing factor (Choi et al., 2005). Moreover, *A. baumannii*'s OmpA enables serum resistance by neutralizing factor H of the complement pathway, thus avoiding complement-mediated killing (Kim et al., 2016; Schweppe et al., 2015; Kim S. W. et al., 2009).

Furthermore, recently discovered porins, such as Omp34 (previously known as Omp33-36), act as fibronectin-dependent porins (Smani et al., 2012b), activating caspases to attach to and injure epithelial cells in the lungs, leading to apoptosis (Rumbo et al., 2014; Smani et al., 2013).

Another porin, CarO, facilitates carbapenem resistance in *A. baumannii* species and reduces the expression of nuclear factor-κB (NF-κB) pathway genes, weakening the host's immune response to the bacterium and promoting bacterial persistence (Sato et al., 2017; Zhang et al., 2019).

##### 3.2.1.2 Pili

Pili, also known as fimbriae, play a crucial role in the motility of *A. baumannii*, as the bacterium lacks flagella. These pili enable the bacterium to perform twitching or surface-associated motility, which is essential for its ability to colonize surfaces and spread within its environment (Yao et al., 2023). Specifically, twitching movement is mediated by type-IV pili, which also play a role in DNA uptake (Corral et al., 2021). Additionally, pili function as virulence factors in *A. baumannii*. Specific pili, such as photoregulated type-I chaperone–usher pili and Csc pili, are responsible for biofilm formation and adhesion, ultimately inducing apoptosis of epithelial cells (Wood et al., 2018; Chen et al., 2022).

##### 3.2.1.3 Capsule (capsular polysaccharides), lipooligosaccharide, and lipopolysaccharides

Lipopolysaccharides (LPS), lipooligosaccharides (LOS), and the capsule are all synthesized in the cytoplasm and later translocated to the outer bacterial surface (Morris et al., 2019).

LPS consists of three parts: an O-antigen repeat, the carbohydrate core, and a lipid-A anchor (Asif et al., 2018). In contrast, LOS lacks the O-antigen repeat and has an extended carbohydrate core (Whitfield and Trent, 2014; Powers and Trent, 2018).

LPS acts as a chemotactic agent that recruits inflammatory cells and induces them to release cytotoxic materials such as IL-6, TNF-α, IL-1β, and IL-8, primarily due to the presence of lipid A, which is immune-stimulatory (Powers and Trent, 2018). Although LOS biosynthetic genes are essential for *A. baumannii*'s survival and prevention of toxic intermediate accumulation, *A. baumannii* can survive without the lipid-A component. Strains lacking lipid-A primarily stimulate toll-like receptor 2 rather than toll-like receptor 4, possibly due to increased outer membrane lipoprotein exposure (Moffatt et al., 2010, 2013).

*A. baumannii* bacteria containing LOS instead of LPS modify the lipid-A moiety to confer antimicrobial resistance (Boll et al., 2015).

The capsule functions as a protective barrier on the extracellular surface, providing protection from cationic antimicrobial peptides and preventing complement activation in about 33% of species with a capsule, thereby delaying phagocytosis (Kaplan et al., 1985). The highly variable capsule loci in *A. baumannii*'s genome allow for flexibility in adaptation (Geisinger and Isberg, 2015; Hu et al., 2013).

The BfmRS two-component regulatory system negatively regulates the formation of the capsule in response to environmental stimuli, including specific antibiotics such as chloramphenicol and erythromycin. Increased expression of the capsule in *A. baumannii* is associated with higher virulence and pathogenicity, potentially complicating treatment and increasing antimicrobial resistance (Geisinger and Isberg, 2015; Chin et al., 2018).

##### 3.2.1.4 Phospholipase

Phospholipases are enzymes responsible for the hydrolysis of phospholipids. *A. baumannii* bacteria possess two types of phospholipases: phospholipase C (PLC) and phospholipase D (PLD; Fournier and Richet, 2006).

These virulence factors exhibit different subtypes, with two PLC and three PLD enzymes encoded by the *A. baumannii* genome (Stahl et al., 2015; Fiester et al., 2016). Both PLC and PLD enzymes affect the stability of the epithelial cell membrane (Lee et al., 2017) and induce erythrocyte hemolysis by exerting cytotoxic effects, aiming to increase the ability to acquire iron from the environment (Morris et al., 2019).

A notable difference between the two types of phospholipases is the conservation of *plc* genes among different *A. baumannii* strains (Stahl et al., 2015; Jacobs et al., 2010). Inactivation of any two *plc* genes leads to a decrease in the cytotoxic effect on host cells (Fiester et al., 2016). On the contrary, the function of *pld* is more specific to bacterial resistance to antibodies and epithelial cell infectivity (Stahl et al., 2015; Jacobs et al., 2010). Deletion of *PLD* genes, including the more significant *PLD2*, only results in a partial decrease in virulence (Stahl et al., 2015).

##### 3.2.1.5 Protein secretion systems

**Figure 3:**
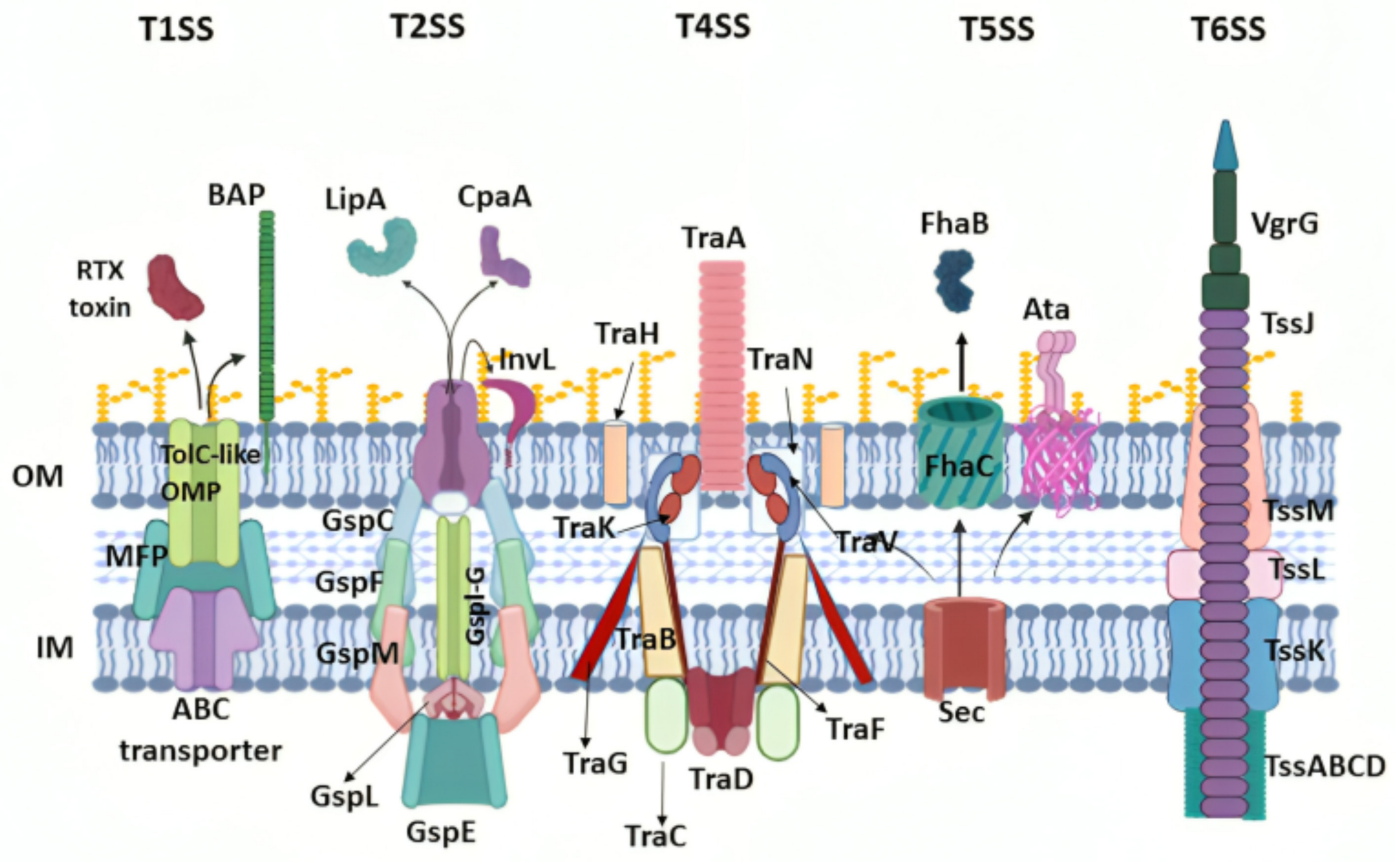
*Acinetobacter baumannii* virulence associated with secretion systems and released effector proteins. The names of the main protein components for each secretion system are indicated, and outer membrane vesicles (OMVs) are also shown. OM, outer membrane; IM, inner membrane. Created in BioRender. ezz, z. (2025) https://BioRender.com/c5l6dl2.

Five secretion systems have been found in *A. baumannii*, where they function to allow the production of proteins responsible for heterogeneous cellular functions (Figure 3).

###### 3.2.1.5.1 Type-I secretion system

Type-I secretion system (T1SS) is a tripartite system composed of an inner membrane adenosine 5'-triphosphate (ATP)-binding protein, a periplasmic adaptor, and an outer membrane pore (Yao et al., 2023). This system delivers proteins from the cytosol to the extracellular environment (Morris et al., 2019), particularly the RTX protein and Bap, which play a major role in attachment to epithelial cells of the respiratory and urinary tract, along with biofilm formation and persistence (Noto et al., 2015; Skerniškyte et al., 2019; Harding et al., 2017).

###### 3.2.1.5.2 Type-II secretion system

Since it consists of a complex of proteins, the type-II secretion system (T2SS) is structurally similar to type-IV pili (Korotkov et al., 2012). T2SS is responsible for transporting proteins from the periplasmic space to the outer cell membrane or extracellular space (Korotkov et al., 2012; Harding et al., 2016). This translocation occurs in two steps: first, proteins are moved to the periplasm and then secreted through T2SS (Korotkov et al., 2012). The significance of this secretion system lies in the types of proteins it transports, including lipase A and H, which break down long-chain fatty acids (Harding et al., 2016; Johnson et al., 2015), and the γ-glutamyl transferase enzyme (GGT), which induces apoptosis, inhibits CD4+ T cells, and enhances colonization by resisting antibiotics (Geisinger et al., 2019; Elhosseiny and Attia, 2018; Elhosseiny et al., 2019, 2020).

###### 3.2.1.5.3 Type-IV secretion system

The type-IV secretion system (T4SS) in *A. baumannii* mediates the conjugative transfer of DNA, plasmids, and additional mobile genetic elements, which is critical for spreading drug-resistant genes among organisms, particularly the *OXA-23* gene (Ayoub Moubareck and Hammoudi Halat, 2020; Liu et al., 2014; Smith et al., 2013).

###### 3.2.1.5.4 Type-v secretion system

Type-V secretion system (T5SS) is the most common and basic secretion system in Gram-negative organisms (Henderson et al., 2000). *A. baumannii* possesses only two of the five known subdivisions of T5SS (Bentancor et al., 2012a,b), namely, the type-Vb and type-Vc secretion systems (Elhosseiny and Attia, 2018; Pérez et al., 2017).

T5bSS includes the proteins AbFhaB, which allows adherence to integrin along with fibronectin, and AbFhaC, which facilitates the recognition and translocation of AbFhaB to the bacterial surface (Elhosseiny and Attia, 2018; Pérez et al., 2017). Moreover, research conducted in murine models indicated that the loss of Vb did not wholly diminish the virulence of *A. baumannii* (Pérez et al., 2017). T5cSS, which is also known as ATA, forms a trimeric autotransporter that facilitates binding to the ECM and basement membrane proteins, including collagen types I–V and laminin. Studies have revealed that the deletion of Ata reduces *Acinetobacter*'s ability to form biofilms and significantly weakens bacterial virulence (Bentancor et al., 2012a).

###### 3.2.1.5.5 Type-VI secretion system

Type-VI secretion systems (T6SS) are activated in *A. baumannii* under stress conditions, such as environmental nutrient depletion, bacterial damage, or competition from other bacteria (Yao et al., 2023; Hood et al., 2017). In *A. baumannii*, the T6SS specifically damages competing bacteria by secreting toxins, which may include peptidoglycan hydrolases, nucleases, or those targeting cell membranes (Morris et al., 2019; Elhosseiny and Attia, 2018; Fitzsimons et al., 2018). Studies have shown that in immunocompromised patients, *A. baumannii*'s T6SS bactericidal activity increases, enabling this opportunistic pathogen to thrive without interference from other bacteria in the low-immunity state (Repizo, 2017).

###### 3.2.1.5.6 Efflux systems

Efflux systems in *A. baumannii*, composed of bacterial efflux pumps (Morris et al., 2019), function alongside the bacterial capsule to respond to envelope stress (Damier-Piolle et al., 2008) by extruding toxic compounds and providing antibiotic resistance (Du et al., 2018). The inner membrane efflux pumps work in conjunction with OmpA to prevent antibiotics from exerting their effects in the periplasmic space (Smani et al., 2014). Six families of efflux pumps have been described in *A. baumannii*, including resistance nodulation cell division family (RND), small multidrug-resistance superfamily (SMR), ATP-binding cassette (ABC) family, major facilitator superfamily (MFS), multidrug toxic compound extrusion family (MATE), and Proteobacterial antimicrobial compound efflux family (PACE). Among these, four families of efflux pumps are associated with antimicrobial resistance in *A. baumannii* (Lin et al., 2014): RND, with the AdeABC efflux pump playing a significant role in resistance, SMR, MFS, and MATE.

###### 3.2.1.5.7 Outer membrane vesicles

OMVs are nano- and sphere-shaped structures ranging from 10 to 300 nm and are produced by all Gram-negative bacteria, including *A. baumannii* (Roier et al., 2016). These vesicles are enriched with various cytoplasmic components, including proteases, LPS, phospholipids, and OmpA (Kwon et al., 2009), as well as superoxide dismutases, which they transport to host cells (Asif et al., 2018; Ellis and Kuehn, 2010). OMVs play a crucial role in bacterial communication, horizontal gene transfer, host-pathogen interactions, and immune evasion. A significant function of OMVs is their contribution to biofilm formation, which enhances *A. baumannii*'s persistence in hostile environments and facilitates antibiotic resistance. Studies have shown that OMVs contain biofilm-associated proteins and polysaccharides that promote bacterial aggregation and adherence to surfaces, creating a foundation for robust biofilm development. Additionally, OMVs influence the structural integrity of biofilms by delivering extracellular DNA and enzymes that modify the biofilm matrix, enhancing its resilience against antimicrobial agents.

Beyond biofilm formation, OMVs intensify the immune response during *A. baumannii* infections, leading to exacerbated inflammation and cellular damage in lung tissue (Nho et al., 2015). A study by Li et al. demonstrated that *A. baumannii* strains producing more OMVs, particularly those containing higher concentrations of virulence factors, induced a stronger immune response and exhibited more significant pathogenic potential (Li et al., 2015). This suggests that OMVs facilitate colonization and contribute significantly to the bacterium's ability to evade immune clearance and establish persistent infections.

##### 3.2.1.6 Penicillin-binding protein (PBP) 7/8 (PBP7/8) and β-lactamase PER-1

The *pbpG* gene encodes PBP7/8, a virulence factor of *A. baumannii*. The loss of this gene renders the pathogen vulnerable to the host's immune system *in vitro* and *in vivo* (Russo et al., 2009).

Another significant virulence factor is the β-lactamase PER-1, known for its extended spectrum compared to common β-lactamases. Strains producing PER-1 demonstrate strong cell adhesion, a trait lacking in PER-1-negative strains (Sechi et al., 2004). Therefore, PER-1 plays a crucial role in the cell-to-cell adhesion of *A. baumannii*.

#### 3.2.2 Nutrient (metal) acquisition systems

##### 3.2.2.1 Iron acquisition (mainly by siderophores)

**Figure 4:**
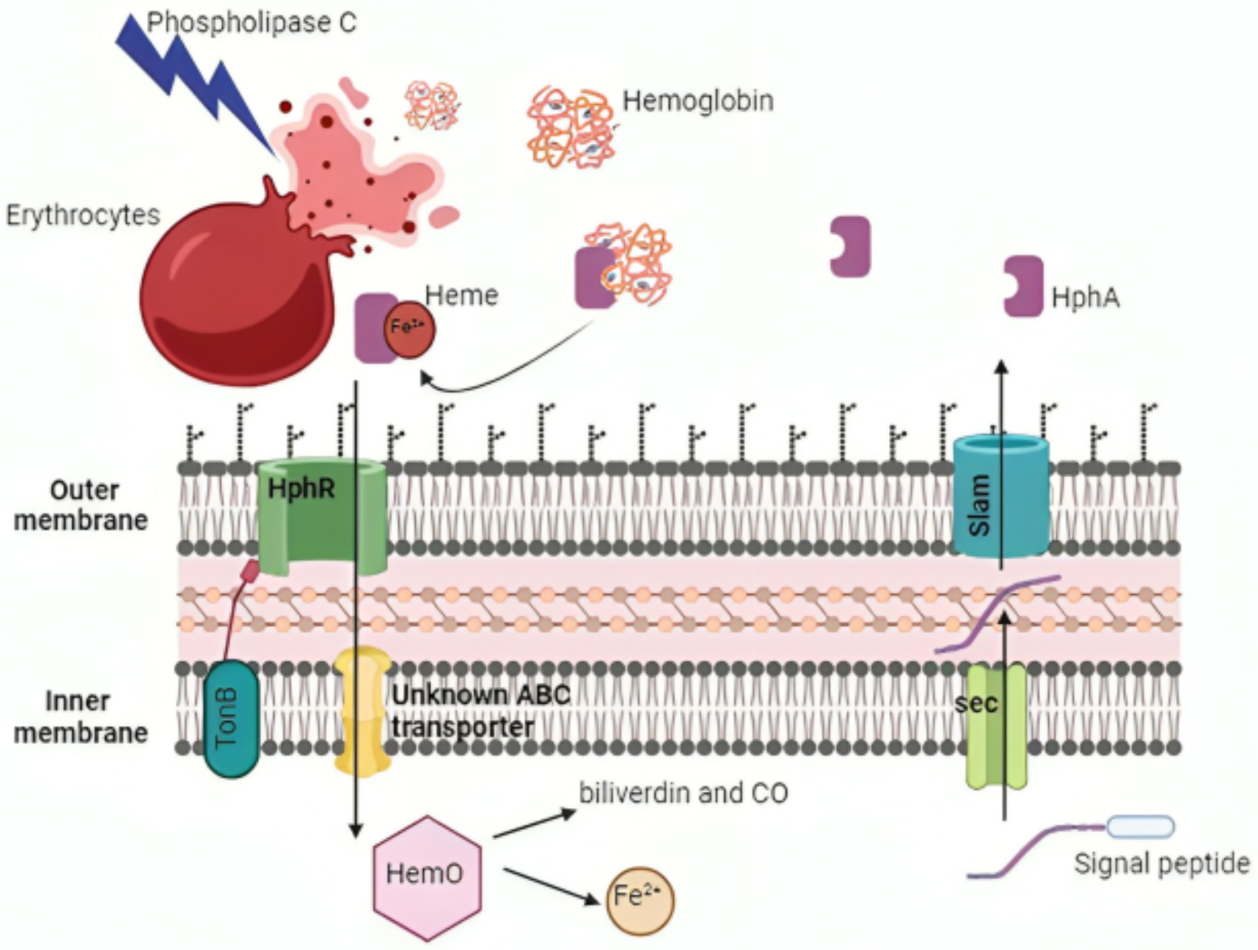
Mechanisms of iron acquisition and utilization by *Acinetobacter baumannii*. *A. baumannii* employs various strategies to obtain and utilize iron from the host. HphA is transported across the inner membrane into the periplasm via the Sec translocon and is then secreted into the extracellular medium by Slam (highlighted in blue). The bacterium's phospholipase C lyses host erythrocytes to extract heme, which is transported into the cell through the TonB-dependent HphR outer membrane receptor. The enzyme HemO liberates iron from heme for cellular use. Created with BioRender. Created in BioRender. ezz, z. (2025) https://BioRender.com/ies2m3c.

**Figure 5:**
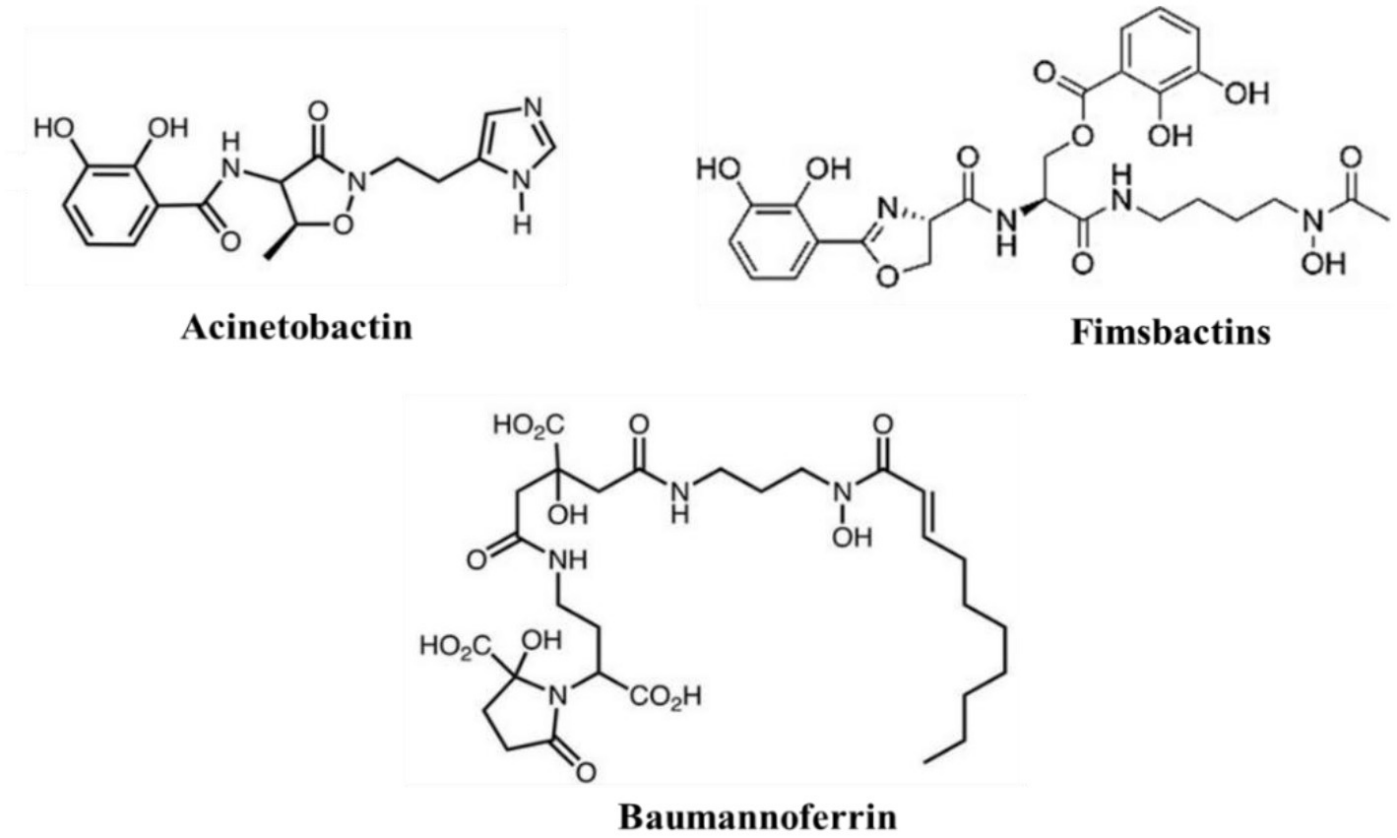
*Acinetobacter baumannii* siderophores: acinetobactin, fimsbactin, and baumannoferrin.

Access to iron in the human body is highly restricted due to the limited solubility of active ferric iron in aerobic environments and chelation by molecules, such as hemoglobin, transferrin, and lactoferrin (Antunes et al., 2011b; Ferreira et al., 2016). To overcome this limitation, *A. baumannii* employs two iron scavenging mechanisms: direct uptake via receptors and transporters, or secretion of high-affinity iron chelator proteins known as siderophores (Eijkelkamp et al., 2011; De Léséleuc et al., 2014; Figures 4, 5). Among these, *A. baumannii* produces multiple siderophores, with acinetobactin particularly essential for virulence and infection. The absence of acinetobactin has been shown to impair the growth of *A. baumannii*, highlighting its critical role in bacterial survival and pathogenicity (Sheldon and Skaar, 2020; Bohac et al., 2019). Additionally, iron-rich environments have been observed to be crucial to promote increased expression of OmpA, a key virulence factor, further emphasizing the importance of iron acquisition in the pathogenesis of *A. baumannii* (Runci et al., 2019; Liu et al., 2021).

##### 3.2.2.2 Zinc and manganese acquisition

*A. baumannii* relies on the import of zinc, crucial for its survival, through the transcription of the zinc uptake regulator (*Zur*) gene, which activates the ZnuABC transporter and ZigA GTPase. Additionally, the bacterium can export zinc via HutH activation and histidine catabolism, promoting zinc release by the *ZigA* gene (Mortensen et al., 2014; Nairn et al., 2016).

Similarly, manganese is another essential nutrient for *A. baumannii*, acquired through analogous mechanisms. Disruption of manganese abundance can impair bacterial colonization in the respiratory tract of the lungs (Juttukonda et al., 2016).

#### 3.2.3 Community interactions

##### 3.2.3.1 Quorum sensing

*A. baumannii* communicates intercellularly with neighboring cells, including other bacteria, by sensing changes in environmental conditions and responding accordingly (Bose and Ghosh, 2016). This communication is facilitated through the secretion of autoinducers, enabling the bacterium to gauge population density and environmental cues for adaptive responses. *A. baumannii* employs hormone-like molecules known as acyl-homoserine lactones as signaling molecules for both inter-species and intra-species communication, facilitating efficient biofilm formation through the expression of *abaI* and *abaR* genes (Niu et al., 2008), as well as regulating motility (Abraham, 2016; Bhargava et al., 2010; Rutherford and Bassler, 2012).

##### 3.2.3.2 Biofilm formation

Biofilm formation is a critical survival strategy for *A. baumannii*, enabling it to thrive in harsh environmental conditions and develop resistance to antimicrobial agents (Mea et al., 2021). This protective process occurs in three distinct phases, including early development, matrix formation, and maturation (Hoyle and Costerton, 1991).

- **Early Development:** Initial adhesion of bacterial cells to surfaces.
- **Matrix Formation:** Production of extracellular polymeric substances, facilitating the formation of structured communities.
- **Maturation:** Formation of a fully developed biofilm with embedded bacterial populations, capable of withstanding host immune responses and antimicrobial agents.

Interestingly, *A. baumannii* strains capable of producing biofilms exhibit twice the survival rate under conditions of desiccation and nutrient depletion (Hoyle and Costerton, 1991). This is particularly concerning in healthcare settings, as biofilms commonly colonize medical devices such as catheters, endotracheal tubes, and prosthetic implants, leading to persistent infections (Gaddy and Actis, 2009). Moreover, biofilm-forming *A. baumannii* strains exhibit enhanced adherence to biomedical materials such as titanium and polystyrene, complicating infection control measures in clinical settings (Loehfelm et al., 2008).

Multiple factors contribute to biofilm formation, including Csu type-1 chaperone-usher pili, which are essential for early-stage biofilm development, promoting bacterial attachment and aggregation (Tomaras et al., 2003), the ATA, which mediates the tight adhesion of bacterial cells within the biofilm (Choi et al., 2009), and Bap and BLP, which regulate interbacterial interactions and surface attachment (De Gregorio et al., 2015).

One of the primary challenges in treating *A. baumannii* infections is the limited penetration of antibiotics into biofilms. The dense extracellular matrix acts as a physical barrier, preventing effective drug diffusion, while embedded bacteria remain in a low metabolic state, reducing their susceptibility to antibiotics that target actively growing cells (Greene et al., 2016). Additionally, biofilms provide an immune-privileged niche, shielding *A. baumannii* from host immune responses and enabling long-term persistence within hospital environments.

Given the pivotal role of biofilm formation in *A. baumannii's* pathogenicity, understanding its regulatory mechanisms is essential for developing targeted therapeutic interventions, such as biofilm-disrupting agents and anti-adhesive surface coatings for medical devices.

### 3.3 Metal acquisition

*A. baumannii* can modify its metabolic and nutritional requirements to adapt to the adverse host environment. *A. baumannii*, like other organisms, requires nutritional metals for survival. These necessary metals are generally iron, zinc, manganese, copper, magnesium, and nickel. They are co-factors in many basic cellular functions. Furthermore, *A. baumannii* exhibits broad tissue tropism and has evolved ways for acquiring nutritional metals in a variety of host habitats.

#### 3.3.1 Iron

*A. baumannii*, like other bacterial pathogens, relies on iron acquisition mechanisms as crucial virulence traits to ensure its survival in a host. Almost all life on Earth depends on iron as a nutrient (Ilbert and Bonnefoy, 2013). It is necessary for the electron transport chain to produce energy as well as other metabolic activities, including enzyme activation of reactions, DNA replication, repair, and gene expression (Caza and Kronstad, 2013). Iron is typically a vital cofactor for bacterial enzymes involved in these processes, such as catalases, succinate dehydrogenases, and cytochromes (Jakubovics and Jenkinson, 2001). Additionally, it is necessary for ribonucleotide reductases that are involved in DNA replication, including NrdAB (Martin and Imlay, 2011). Given that *A. baumannii* has a nutritional need for iron, acquiring it from the iron-limited host environment is essential for the infection to progress (Frawley and Fang, 2014). This occurs through different mechanisms and strategies, including:

##### 3.3.1.1 The uptake of heme via erythrocyte lysis

Many *A. baumannii* strains can get heme-bound iron, despite the fact that the majority of the iron in the human host is attached to heme groups inside hemoglobin molecules. Hemolytic proteins allow *A. baumannii* to reach iron pools within erythrocytes (Figure 4), and are encoded by hemolysin-related genes and two PLC genes (*plc1* and *plc2*; Antunes et al., 2011a; Fiester et al., 2016). In a study by Fiester et al. (2016), horse erythrocytes coincubated with *A. baumannii* grown in iron-chelated media showed considerable cell lysis and damage to the cell membrane, but not when grown in iron-rich medium. The genes *plc1 and plc2* may be implicated in the lysis of erythrocytes to obtain iron, as evidenced by their overexpression and correlation with iron limits. The absence of these genes in non-pathogenic *Acinetobacter baylyi* ADP1 strains, but their presence in all sequenced *A. baumannii* genomes, raises the possibility that *plc1 and plc2* gene products play a role in virulence (Fiester et al., 2016).

Heme uptake systems are used by *A. baumannii* to extract iron from heme after lysing erythrocytes (Runyen-Janecky, 2013; Figure 4). Two heme uptake gene clusters were identified by genome analysis of clinical isolates of *A. baumannii* (Giardina et al., 2019). A periplasmic heme-binding protein, an inner membrane ABC transporter, and a TonB-dependent outer membrane receptor are all encoded by heme uptake cluster 1. A putative heme oxygenase (HemO), an extracytoplasmic function (ECF) sigma factor and its anti-sigma factor, and a TonB-dependent receptor are all encoded by heme uptake cluster 2. HphA, a Slam protein, is a crucial hemophore that the cell secretes throughout the heme import process (Bateman et al., 2021). It has two purposes: it binds hemoglobin and scavenges free heme (Bateman et al., 2021). A two-component receptor system that incorporates hemoglobin-bound iron into *A. baumannii* cells is formed when HphA transfers these iron sources to the HphR outer membrane receptor. For *A. baumannii* to reach its maximum virulence, this heme absorption mechanism is necessary (Bateman et al., 2021).

The heme oxygenase (abHemO) has been revealed to be necessary for heme absorption and utilization (Giardina et al., 2019). It has been demonstrated that in other organisms, HemO catalyzes the oxidative cleavage of heme to biliverdin and CO to liberate iron (Ratliff et al., 2001). In contrast to *A. baumannii* strain ATCC 17,978, which encodes fimsbactin siderophore gene clusters in place of *hemO*, one study showed that *A. baumannii* LAC-4, a hypervirulent and hyper-resistant strain, can effectively utilize iron bound to heme (Giardina et al., 2019). Western blotting revealed that LAC-4 was able to express the AbHemO protein and proliferate when given heme as the only iron source. The growth curves of ATCC 17,978 produced with heme as the only iron supply resembled those observed under low-iron circumstances, suggesting that this strain is unable to utilize the extracellular heme (Giardina et al., 2019).

Furthermore, the substrates that HemO can convert to biliverdin were examined in the same study. Extracellular heme served as the only source of iron for *A. baumannii* cells during growth. Isotope labeling of carbon allowed for the separation of extracellular heme from intracellular heme and for quantifying the relative amounts of each heme type in the medium. The findings supported HemO's uptake and metabolism of extracellular heme, and any intracellular heme discovered in the medium was assumed to be the consequence of heme turnover brought on by the preservation of iron homeostasis (Giardina et al., 2019).

##### 3.3.1.2 Siderophores

**Figure 6:**
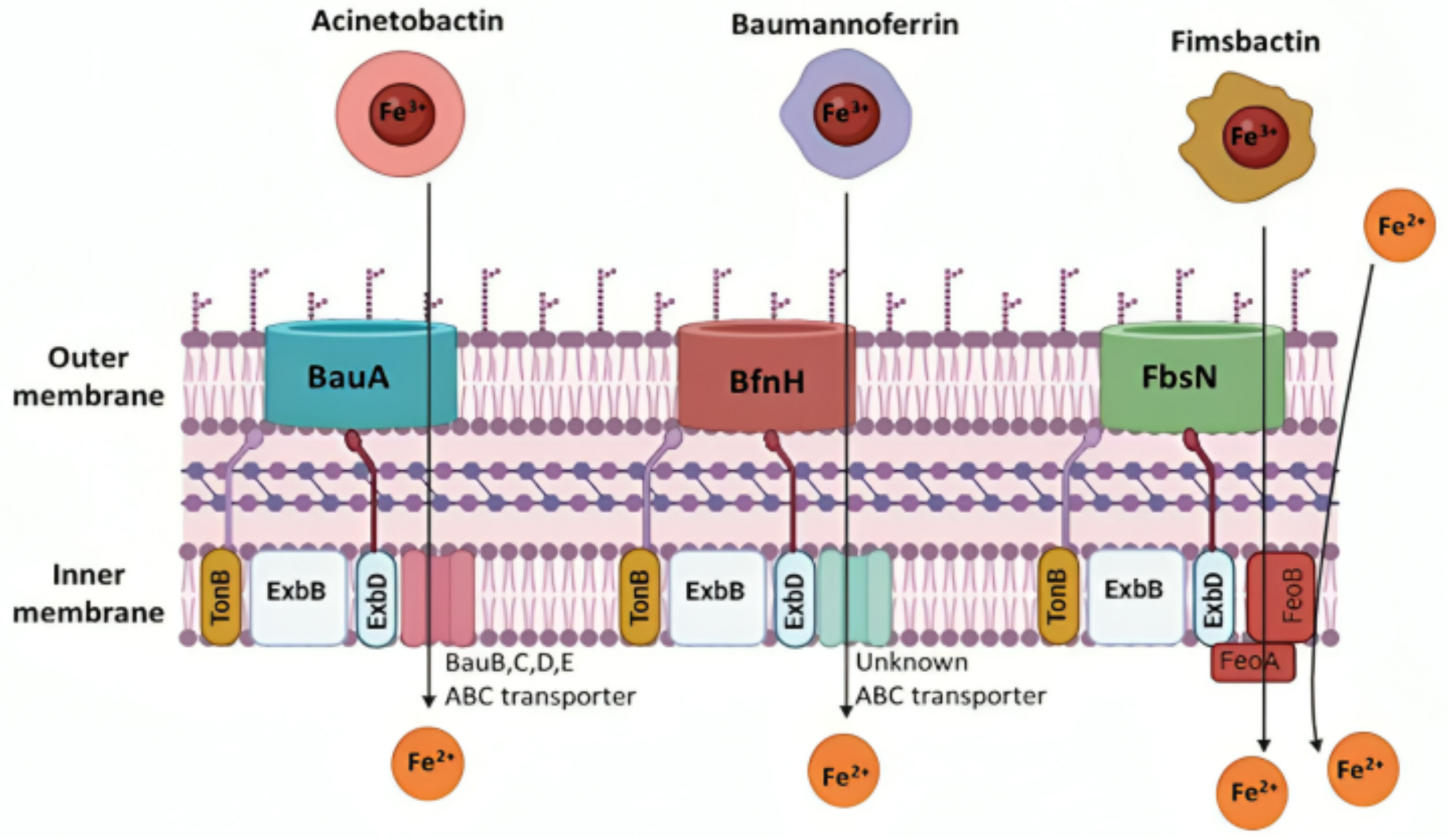
Iron transport mechanisms of *Acinetobacter baumannii* siderophores. The siderophore Acinetobactin has a high affinity for iron and utilizes an inner membrane ATP-binding cassette (ABC) transporter, consisting of BauB, BauC, BauD, and BauE, along with the BauA TonB-dependent outer membrane receptor, driven by proton-motive force. Baumannoferrin also exhibits a strong affinity for iron, transporting it through an inner membrane ABC transporter and the BfnH TonB-dependent outer membrane receptor, both using proton-motive force. Similarly, Fimsbactin transports iron using the FeoA transporter and the FbsN TonB-dependent outer membrane receptor, with both mechanisms relying on proton-motive force. The cytoplasmic membrane's FeoAB is essential for importing ferrous iron (Sheldon and Skaar, 2020). Created in BioRender. ezz, z. (2025) https://BioRender.com/pels3ql.

Siderophores are secondary metabolites secreted during the iron-acquiring process. They are advantageous because they permit iron to be scavenged extracellularly (Khasheii et al., 2021). Usually, iron-loaded siderophores bind a receptor and gain access to the bacterial cell (Paquelin et al., 2001). After additional processing, the iron is extracted and used as needed for vital physiological functions (Page, 2019). In *A. baumannii*, iron chelators siderophores are classified into three types: Acinetobactins, fimsbactins, and baumannoferrins (Figure 6; Page, 2019; Song and Kim, 2020). When iron levels are low, all three siderophore gene clusters are elevated, and siderophore synthesis is suppressed when more iron is added to the medium. The increase in gene expression caused by zinc depletion is 10–1,000-fold smaller than that caused by iron depletion; even so, this slight change in expression may indicate that, while siderophores are primarily iron-regulated, zinc can also partially regulate them in situations where metal constraints are sustained (Conde-Pérez et al., 2021; Sheldon and Skaar, 2020). Because studies have shown that deletion of one siderophore system does not significantly alter total siderophore activity in strains with many classes, it is assumed that the various siderophores have some redundancy. Cells can be sufficiently capable of chelating iron *in vitro* by expressing just one of these siderophore systems (Sheldon and Skaar, 2020).

###### 3.3.1.2.1 Acinetobactin

Clinical isolates of *A. baumannii* have a high degree of conservation in the versatile metal siderophore acinetobactin. Two isomers of acinetobactin exist: one is more active at neutral and basic pH levels, while the other is more active at acidic pH levels (Kim et al., 2021). All the genes in the acinetobactin gene cluster were revealed to be substantially elevated in low-iron environments using transcriptional profiling (Eijkelkamp et al., 2011). Research has demonstrated that the acinetobactin siderophore plays a significant role in iron uptake from host serum proteins such as lactoferrin and transferrin (Sheldon and Skaar, 2020). When cultivated in metal-chelated minimum medium containing human serum, mutants missing acinetobactin were seen to develop less rapidly than the fimsbactin and baumannoferrin mutants under the same conditions. The observed decreased growth was comparable to what was observed when the mutants were treated on human transferrin as their only source of iron (Sheldon and Skaar, 2020). A mouse infection model demonstrated the significance of acinetobactin in *A. baumannii* pathogenesis, as it was the sole siderophore determined to be critical for pathogenesis (Sheldon and Skaar, 2020). Acinetobactin plays a crucial role in the virulence of *A. baumannii*, as evidenced by the fact that it is the only expressed siderophore system and that it can survive in iron-limiting environments in both vertebrate and invertebrate hosts (Penwell et al., 2012).

Acinetobactin is required for intracellular *A. baumannii* infection, according to a study that used human alveolar epithelial cells. This was shown by the fact that acinetobactin mutant strains exhibited much lower intracellular persistence as compared to the wild-type ATCC 19,606 (Gaddy et al., 2012). Once more, alveolar epithelial cells were infected with these strains to track the rates of apoptosis in human cells. Compared to the wild type, acinetobactin mutants showed a 2-fold decrease in apoptosis rates, whereas acinetobactin receptor mutants showed a 24-fold decrease in apoptosis rates. This indicates that the acinetobactin siderophore system-expressing *A. baumannii* causes greater cell damage. The acinetobactin system is necessary for *A. baumannii*'s pathogenicity in *Galleria mellonella* and mouse models, according to these infection assays (Gaddy et al., 2012).

Certain commensal bacteria may also be inhibited by acinetobactin. Knauf et al. observed that all *Staphylococcus* species and *Corynebacterium striatum* growth were considerably suppressed when co-plating *A. baumannii* ATCC 17,978 with common skin and nose commensals in iron-poor environments (Knauf et al., 2022). Acinetobactin production and transport genes were shown to make up a significant fraction of the genes implicated in the suppression of the *Staphylococcus* epidermidis library, with the use of a transposon mutant library utilizing *A. baumannii* AB5075 (Knauf et al., 2022). In comparison to wild-type *A. baumannii*, acinetobactin mutants also showed decreased suppression of *Staphylococcus hominis* and *C. striatum*. Acinetobactin is believed to be the primary siderophore responsible for this reported impact. However, other siderophores may also be involved in commensal suppression through iron competition (Knauf et al., 2022).

###### 3.3.1.2.2 Baumannoferrin

Studying *A. baumannii* AYE led to the discovery of baumannoferrin. A mutation in *entA* prevented this strain from producing acinetobactin. Outside of the acinetobactin gene cluster, the *entA* gene plays a crucial role in the production of 2,3-dihydroxybenzoic acid, which is a prerequisite for acinetobactin. Even though *A. baumannii* AYE was unable to make acinetobactin; it was still able to thrive in low-iron environments (Gaddy et al., 2012). Upon closer inspection of *A. baumannii* clinical isolates, three gene clusters encoding siderophores were discovered. Acinetobactin was found to be encoded by one of these gene clusters. In contrast, a hydroxamate siderophore was found to be conserved in all of the isolates that were analyzed, including *A. baumannii* AYE (Antunes et al., 2011a). When it was discovered that elements of this putative gene cluster were increased in low-iron environments, it was assumed that the cluster—now known as baumannoferrin—was involved in iron acquisition (Eijkelkamp et al., 2011). According to baumannoferrin's characterization, this iron chelator comprises comprises citrate, decenoic acid, 1,3-diaminopropane, 2,4-diaminobutyrate, and α-ketoglutarate make up this iron chelator. It was discovered that baumannoferrin has two isomers: baumannoferrin A and baumannoferrin B, which are only different by one double bond. Genes required for baumannoferrin's production, transport, and intracellular uptake are found in the gene cluster encoding it (Penwell et al., 2012). It is believed that baumannoferrin is the only siderophore that *A. baumannii* AYE uses, demonstrating that it is adequate on its own for survival in conditions lacking in iron (Penwell et al., 2012). The virulence of *A. baumannii* AYE in *G. mellonella* is similar to that of other clinical isolates of *A. baumannii*, indicating that *A. baumannii* AYE does not require acinetobactin for pathogenesis. In comparison, *A. baumannii* ATCC 19,606 encodes all three siderophores, but to be pathogenic against *G. mellonella* and in mice, it still needs a functional acinetobactin system (Penwell et al., 2012).

###### 3.3.1.2.3 Fimsbactins

A third class of siderophores called fimsbactins is present in fewer than 10% of sequenced *A. baumannii* strains (Bohac et al., 2019). Among these strains that possess fimsbactin are *A. baumannii* 6,013,150 and *A. baumannii* ATCC17978 (Proschak et al., 2013). Fimsbactins consist of one hydroxamate and two catecholates. Their iron-chelating motifs include putrescine and L-serine/threonine in their backbone. The principal siderophore is fimsbactin A, whereas fimsbactins B through F are assumed to be intermediates in the biosynthesis (Bohac et al., 2019). In an investigation aimed at demonstrating the siderophore properties of fimsbactin, *A. baumannii* ATCC 17,978 was cultured in a low-iron medium, enabling very slow development. It was observed that in a dose- and time-dependent manner, both fimsbactin alone and fimsbactin loaded with ferric iron enhanced cell proliferation (Bohac et al., 2019). Interestingly, this same study found that adding fimsbactin A counteracted the growth improvements brought about by the exogenous acinetobactin siderophore to the media. Pathway competition is a potential explanation for the antagonistic effect found (Bohac et al., 2019). The non-ribosomal peptide synthetase (NRPS) assembly systems are used to create both fimsbactin and acinetobactin. They sharethe precursors of L-threonine and 2,3-dihydroxybenzoic acid (DHBA; Proschak et al., 2013). Furthermore, it was discovered that the BauB periplasmic siderophore-binding protein (Bohac et al., 2019) is directly competed with by these two siderophores for binding. Conde-Pérez et al. investigated the acinetobactin and fimsbactin gene clusters. They discovered that every acinetobactin gene, except *entA*, has a possible redundant equivalent in the fimsbactin gene cluster (Conde-Pérez et al., 2021). This group postulated that fimsbactin would, therefore, serve as a substitute in the event that acinetobactin is rendered inactive, thereby elucidating the reason for the relative rarity of fimsbactin among isolates of *A. baumannii* (Conde-Pérez et al., 2021). Further research is necessary to determine the implications of the redundancy between acinetobactin and fimsbactin and any possible connections between baumannoferrin and these two siderophores.

###### 3.3.1.2.4 Siderophores transport

The active process of siderophore- or heme-bound ferric iron transport over the outer membrane and into the periplasmic space is powered by the proton-motive force of the TonB-ExbB-ExbD protein complex (Figure 6; Noinaj et al., 2010). Through a brief transmembrane N-terminal region, TonB binds to ExbB and ExbD (Klebba, 2016). *In vitro* complex formation between TonB and the outer membrane protein FhuA requires a proline-rich spacer inside the TonB structure (Postle and Larsen, 2007). However, TonB function *in vivo* is unaffected by the removal of this spacer (Seliger et al., 2001). The presence of this outer membrane protein and the TonB complex *in vivo* requires more study.

The genomes of *A. baumannii* have been found to contain about 21 putative TonB-dependent outer membrane transporter genes, some of which are iron-regulated (Antunes et al., 2011b). Three coding genes for the TonB have been found and given the names *tonB1, tonB2*, and *tonB3* (Zimbler et al., 2013). It has been demonstrated that the overexpression of the iron-regulated *tonB3*, which is overexpressed in iron-chelated as opposed to iron-rich medium (Eijkelkamp et al., 2011), is required for *A. baumannii* survival in iron-limiting conditions. It is also important for *A. baumannii* pathogenicity in *G. mellonella* insect models and mouse mammalian models (Eijkelkamp et al., 2011; Runci et al., 2019). Furthermore, *A. baumannii* ATCC 19,606 *tonB1 tonB2* double mutants exhibit much reduced virulence in comparison to the original strain; yet, virulence remains unaffected by inactivating either *tonB1* or *tonB2* on its own (Zimbler et al., 2013). All of these results point to the importance of *tonB3* in the transfer of iron chelators as opposed to the more minor roles that tonB1 and *tonB2* play in this process (Zimbler et al., 2013).

Iron regulation is present in the TonB complex's *tonB3* gene because of its Fur-controlled promoter. Fur binds to ferrous iron to form a repressor complex, which prevents transcription of Fur-controlled promoters when iron levels are high enough. When iron is deficient, apo-Fur binds to the promoter, activating transcription (Fillat, 2014). Additionally, it has been discovered that TonB transporters are more abundant in OMVs (Figure 6; Dhurve et al., 2022). All Gram-negative bacteria produce OMVs, which can transport iron and other nutrients (Dhurve et al., 2022). It was discovered that the OMVs of *A. baumannii* DS002 had 19 distinct TonB transporters. Seven of these transporters had structural characteristics found in other transporters involved in the translocation of siderophore-bound iron, according to an *in silico* study (Dhurve et al., 2022). This implies that OMVs are engaged in ensnaring siderophores from adjacent bacteria for *A. baumannii* to receive ferric iron (Dhurve et al., 2022).

Moreover, under iron-limiting circumstances, only *tonB3* is upregulated among *A. baumannii* TonB systems, suggesting an association with iron homeostasis. Compared to the wild-type, *tonB1, tonB2*, and *tonB1 tonB2* mutants of *A. baumannii* are less efficient at acinetobactin and iron transfer and are unable to grow in iron-starved environments. A *tonB3* mutant has not been created, which may indicate that this gene is required for growth (Zimbler et al., 2013). Many bacteria express ferrous iron uptake systems in a reduced ferrous iron environment, most notably FeoAB transporters, which are required for iron acquisition and pathogenesis. FeoAB and its regulator FeoC are putative ferrous iron import systems encoded by *A. baumannii*, and all sequenced strains have at least one FeoB in addition to FeoA and FeoC (Mortensen and Skaar, 2013). In addition, a study that investigated the entire transcriptional response of *A. baumannii* to iron deprivation revealed that the main characteristic of this transcriptional response was the upregulation of three siderophore-mediated iron acquisition systems. Given the significant degree of overexpression of these systems in the presence of iron limitation, it is likely that each of them plays a crucial role in facilitating iron uptake and is hence essential for A. baumannii to survive in environments with low iron levels, like human hosts. Significant differences in expression were also seen for several genes related to other functions, including respiration and electron transport. They revealed that 463 genes had transcription levels that were more than 2 times higher under low iron conditions, with 95 of those genes having more than 4 times higher transcription levels. Significantly, there was a significant upregulation of three gene clusters related to siderophore production, including one unique cluster. The ferric uptake regulator (Fur) was found to have binding sites in the promoter regions of numerous upregulated genes, indicating that it plays a significant role in the iron acquisition response of *Acinetobacter*. Several motility-related genes were among the genes that were downregulated in response to decreased iron availability (Eijkelkamp et al., 2011). *A. baumannii* also modulates its outer membrane protein composition, with observed changes in proteins like OmpA, to optimize iron uptake. Proteins associated with iron storage (Bfr), energy and metabolic processes (AcnA, AcnB, GlyA, SdhA, and SodB), and lipid biosynthesis are among the many proteins represented by the iron-induced protein spots. Identifying an iron-regulated Hfq ortholog suggests that Fur and short RNAs, as reported in other bacteria, may mediate iron regulation in this bacterium. The reduced capacity of an OmpA isogenic deficient derivative to grow in iron-chelated circumstances indicates that OmpA plays a function in iron metabolism, as suggested by the iron-induced synthesis of this protein (Nwugo et al., 2011).

##### 3.3.1.3 The ferrous transport system FeOAB

In Gram-negative bacteria, the primary mechanism for ferrous iron transport is called Feo (Figure 6) *A. baumannii* strains retain both the FeoAB import mechanism and its regulator, FeoC. The feo operon codes for the cytosolic transcriptional repressor FeoC, the protein responsible for active ferrous iron transport across the cytoplasmic membrane FeoB, and the cytosolic protein FeoA, which has an unclear function. It is interesting to note that *feoB* deletion has no effect on *A. baumannii* growth in iron-poor M9 minimum media, nor does it stop the production of siderophores or reduce *A. baumannii* pathogenicity. Under these circumstances, ferric iron uptake is thought to be more active than ferrous iron transport. Nonetheless, it appears that the Feo system is required to grow *A. baumannii* in human serum that has iron chelated to transferrin. When cultured in human serum, mutants with *feoB* deletion showed a 4-fold decrease in cell density compared to the wild type, indicating that the Feo system may help *A. baumannii* proliferation. When exposed to the complement systems inside normal human serum, *A. baumannii feoB* mutant cells exhibited accelerated cellular aging. They died in significantly higher numbers than wild-type cells. Further research is necessary to clarify the contradicting findings indicating that FeoB is essential for growth in iron-chelated human serum but not in iron-poor minimum media. FeoB was also unexpectedly shown to be substantially increased under iron-poor settings (as opposed to iron-supplemented conditions) using reverse transcription-quantitative polymerase chain reaction (RT-qPCR), even though it was not determined to be necessary for *A. baumannii* growth in minimum medium (Cook-Libin et al., 2022).

Given that putative Fur boxes were found inside the promoters of these genes, the *feo* operon is iron controlled similar to *tonB3*. The Feo system's Fur box is located downstream of the *feoA* promoter's transcriptional start site, further supporting the idea that the Feo system's expression is iron-dependent (Cook-Libin et al., 2022).

#### 3.3.2 Zinc

Although zinc uptake and utilization mechanisms have been studied, non-iron metal homeostasis in *A. baumannii* is not as well understood. An inner membrane ABC zinc transporter termed ZnuABC is preserved and is encoded by *A. baumannii*. Numerous bacterial species, including *Salmonella, E. coli*, and *Y. pestis*, have been shown to contain ZnuABC systems, which have occasionally been shown to be necessary for virulence (Ammendola et al., 2007). The ZnuABC transporter in *A. baumannii* is activated during zinc deficiency and *A. baumannii* pneumonia in mice. The inner membrane permease ZnuB is necessary for both bacterial growth in the mouse pneumonia model and the development under zinc-limiting circumstances. The Fur family Zur, a zinc-sensing repressor that detects a conserved Zur box DNA sequence upstream of target genes when zinc-bound, is also encoded by *A. baumannii*. Genes implicated in intracellular zinc homeostasis, the TonB/ExbB/ExbD system, and potential outer membrane zinc transporters ZnuD_1_ and ZnuD_2_ are among the tentative list of Zur target genes that have been found (Hood et al., 2012). Moreover, when zinc is limited, the expression of *znuA, znuB, znuC, znuD*_1_*, znuD*_2_, and *tonB* increases. No TonB system has yet been proved to be directly engaged in zinc acquisition through energy transmission to TonB-dependent receptors, even though TonB-dependent receptors are involved in zinc acquisition. Further investigation is necessary to elucidate the roles of these proteins and their significance in the many *in vivo* niches of *A. baumannii*. Ultimately, more research is required to determine and describe the bacterial systems involved in the import and use of additional non-iron metals (Mortensen and Skaar, 2013).

In summary, *A. baumannii* employs a multifaceted pathogenic strategy, beginning with adhesion to host surfaces and medical devices via hydrophobic interactions, pili, and biofilm-associated proteins. Its ability to survive for months on abiotic surfaces facilitates nosocomial transmission, while motility mechanisms enhance colonization. Biofilm formation, regulated by quorum sensing and extracellular matrix components, provides resistance to antibiotics and immune clearance. Key virulence factors include OMPs that mediate host cell adhesion, immune evasion, and apoptosis induction. Secretion systems contribute to toxin delivery, biofilm formation, and horizontal gene transfer, while efflux pumps enhance antibiotic resistance. Iron acquisition via siderophores and heme uptake systems is critical for survival in host environments, linking nutrient scavenging to virulence. Together, these mechanisms enable *A. baumannii* to persist in hospitals, evade immune responses, and resist treatment, underscoring its role as a critical opportunistic pathogen.

## 4 Clinical manifestations

*A. baumannii* is one of the most severe MDR pathogens, leading to widely variable clinical manifestations and outcomes (Gordon and Wareham, 2010). The mortality rate can vary depending on the site of infection, which may affect various parts of the body, including the respiratory tract, central nervous system (CNS), urinary tract, skin, and bloodstream. *A. baumannii* is predominantly a nosocomial infection, and multiple studies have identified several risk factors associated with a high risk of infection. These include previous or prolonged stays in the ICU or hospital, advanced age, use of medical devices such as catheters, endotracheal or nasogastric tubes, mechanical ventilation, prior antimicrobial therapy, previous major or invasive procedures, dialysis, prematurity, low birth weight, and prolonged use of parenteral nutrition or intravenous lipids (Djordjevic et al., 2016; Fukuta et al., 2013).

### 4.1 Respiratory tract infections

*A. baumannii* is predominantly found in the respiratory tract, particularly among individuals undergoing mechanical ventilation. Distinguishing upper respiratory tract colonization from verifiable pneumonia can be challenging since this pathogen primarily colonizes the respiratory tract, with minimal skin colonization (Peleg et al., 2008). In Lebanon, *A. baumannii* is most isolated from the respiratory tract (53.1%), followed by the surgical wound (18.8%), blood (15.6%), urine (10.2%), and other sites (2.3%; Kanafani et al., 2018).

As previously discussed, *A. baumannii* is mainly hospital-acquired, as indicated by a recent study showing a transmission rate of 315.4 cases per 1,000 ICU patient days, with a high mortality rate ranging from 52 to 66% (Huang Y. et al., 2019). Ventilator-associated pneumonia (VAP) acquired from MDR *A. baumannii* is associated with a high mortality rate in critically ill patients (Jaruratanasirikul et al., 2019). While *A. baumannii* accounts for 8–14% of VAP cases in the United States and Europe, higher rates are observed in Asia (19% to over 50%), Latin America, and some Middle Eastern countries (Lynch et al., 2017). Nosocomial outbreaks can occur in hospitals due to the colonization of care professionals' hands and inadequate personal hygiene (Peleg et al., 2008). A recent 2-year retrospective study of MDR *A. baumannii* respiratory infections in critically ill patients in Saudi Arabia found that 6.2% of ICU patients developed respiratory infections, with 93% of these cases progressing to VAP. The study also reported a high mortality rate of 74%, with COVID-19 co-infection leading to worse outcomes (Hafiz et al., 2023).

Although *A. baumannii* is primarily a nosocomial agent causing hospital-acquired pneumonia (HAP), community-acquired pneumonia (CAP) is also possible. CAP associated with *A. baumannii* presents an aggressive and sudden course, high bacteremia, and mortality rates, particularly in tropical regions, such as Asia and Australia. CAP is notably linked to the rainy season, excess alcohol consumption (with 10% of individuals being throat carriers), diabetes, smoking, and chronic lung disease (Dexter et al., 2015; Anstey et al., 2002; Wong et al., 2017). A study conducted in China revealed that CAP was linked to a plasmid-encoded bla-OXA-72 with high resistance to carbapenems (Jia et al., 2019).

### 4.2 Meningitis

Meningitis represents the most severe form of *A. baumannii* infection, often occurring post-neurosurgery, and is associated with a high mortality rate, reaching up to 70%, particularly in patients with specific risk factors such as ventriculostomy tubes, prior cerebrospinal fistulae, or antimicrobial therapy following surgery (Siegman-Igra et al., 1993). Mortality rates tend to be higher in cases involving individuals over 40 years old, external ventricular drain usage, elevated white blood cell counts in cerebrospinal fluid, diabetes, and hypertension. The extensively drug-resistant (XDR) phenotype of *A. baumannii* has shown sensitivity only to colistin and tigecycline, as revealed by a large case series in 2019 (Sharma et al., 2019).

A report from China on post-neurosurgical meningitis caused by *A. baumannii* indicated a prevalence of MDR and XDR strains at 33.64%. These strains exhibited high resistance rates, including 100% resistance to imipenem and meropenem, 98.38% to cefazolin, 100% to ceftazidime, 100% to ceftriaxone, and 98.39% to cefepime. However, they demonstrated sensitivity to polymyxin B (100%), tigecycline (60.66%), and amikacin (49.18%; Pan et al., 2018). A recent single-center retrospective study on *A. baumannii* meningitis in children found that mechanical ventilation, septic shock, carbapenem-resistant *A. baumannii* (CRAB), lower peripheral leukocyte counts, along with higher protein levels in the cerebrospinal fluid (CSF) and elevated procalcitonin levels, were significantly associated with failed treatment outcomes (Wang et al., 2024).

Community-acquired meningitis caused by *A. baumannii* is rare and predominantly affects healthy individuals in hot climates, with low rates of drug resistance (Chang et al., 2000). Conversely, nosocomial cases have been associated with the administration of intrathecally contaminated methotrexate and the use of suctioning equipment in neurosurgery units (Kelkar et al., 1989). Clinical features of *A. baumannii* meningitis typically include fever and meningeal signs, with or without seizures. Lumbar puncture often reveals pleocytosis with high neutrophil count, elevated protein levels, and a decreased CSF to serum glucose ratio (Rodríguez Guardado et al., 2001).

### 4.3 Bloodstream infection

VAP and bloodstream infections are prominent clinical manifestations of *A. baumannii* infections. In a study from the United States, *A. baumannii* ranked as the 10th most common cause of all monomicrobial hospital-acquired bloodstream infections (Wisplinghoff et al., 2004). ICU-acquired bloodstream infections were more prevalent than non-ICU infections, with mortality rates ranging from 34 to 43.4% in ICU settings and 16.3% in non-ICU settings. *A. baumannii* is noted for having the third highest mortality rate in ICU settings, following *P. aeruginosa* and *Candida*. It is noteworthy that *A. baumannii* bloodstream infections typically occur after a mean duration of 26 days, making it one of the latest-onset bloodstream infections acquired in the hospital (Wisplinghoff et al., 2004).

Bloodstream infections with *A. baumannii* are frequently associated with vascular catheters and the respiratory tract, with less common associations with skin injuries or urinary tract infections. Mortality rates are higher in pneumonia cases compared to catheter-related infections (Chen et al., 2005; Seifert et al., 1995). Various risk factors contribute to these infections, including cancer, immunocompromised states, burns, prolonged hospital stays, and invasive procedures (Chen et al., 2005; Seifert et al., 1995; Wisplinghoff et al., 2000; Cisneros and Rodríguez-Baño, 2002; García-Garmendia et al., 2001; Gómez et al., 1999; Tilley and Roberts, 1994). Notably, a Brazilian study revealed that 68% of bacteremia cases were linked to cancer patients, with a high mortality rate attributed more to bacterial control than cancer-related effects like low white blood cell count (Freire et al., 2016). In severely burned patients, *A. baumannii* was identified as the most common cause of bacteremia over 4 years, exhibiting 100% resistance to all antibiotics except for low resistance to polymyxin B and minocycline (Gong et al., 2016). Additionally, 102 patients presented with bacteremia among American military personnel injured in Iraq and Afghanistan (Centers for Disease Control Prevention, 2004). Septic shock was reported in one-third of individuals with bacteremia (Seifert et al., 1995; Cisneros et al., 1996).

### 4.4 Urinary tract infection

Urinary tract infections (UTIs) caused by *A. baumannii* are relatively rare, accounting for only 1.6% of all ICU-acquired UTIs (Gaynes and Edwards, 2005). Among these cases, 95% are associated with catheter infections or colonization (Gaynes and Edwards, 2005). Another study has shown that more than 50% of *A. baumannii* strains isolated from urine samples come from catheterized patients, emphasizing its strong association with device-related infections (Mohamed et al., 2022). Moreover, Di Venanzio et al. analyzed *Acinetobacter* isolates from over 19,000 cases in the BJC Healthcare System between 2007 and 2017. The study showed that 17.1% of the cases came from the urinary tract, and only 2% of UTIs are caused by this pathogen (Di Venanzio et al., 2019).

The challenge in managing *A. baumannii*-associated UTIs is compounded by its high resistance to commonly used antibiotics. A retrospective study analyzing 749 urine samples to determine the development of susceptibility to 10 antibiotics and the antibiotic consumption of patients with suspicion of UTI found that *A. baumannii* isolates exhibited high resistance to carbapenems and piperacillin-tazobactam, rendering these agents unsuitable for empirical therapy. Among the tested antibiotics, colistin remained the most active *in vitro*. The study also reported a marked increase in piperacillin-tazobactam consumption, despite its declining efficacy against *A. baumannii* (Jiménez-Guerra et al., 2018). Community-acquired UTIs involving *A. baumannii* are even rarer. In a study conducted across 10 hospitals in Korea, 55.6% of *A. baumannii* strains were implicated in UTIs, with 19.8% exhibiting resistance to imipenem, 25% to meropenem, 13.5% to polymyxin B, and 17.7% demonstrating resistance to colistin (Park et al., 2010).

### 4.5 Skin infections

Skin injuries, burns, and wounds are susceptible to *A. baumannii* infections, particularly in military or disaster settings (Ayoub Moubareck and Hammoudi Halat, 2020). This has been notably reported in retrospective studies of the US military, primarily in Iraq and Afghanistan (Johnson et al., 2007). Injuries infected with *A. baumannii* are often associated with using prosthetic materials, and larval therapy may be considered. While community-acquired or hospital-acquired skin infections caused by *A. baumannii* are rare, they can manifest as cellulitis, necrotizing fasciitis, abscesses, and folliculitis (Glew et al., 1977; Chiang et al., 2003; Ng et al., 2004; Adler et al., 2014). Although more common in war injuries and military service, *A. baumannii* is also implicated in burn units, presenting challenges for elimination (Trottier et al., 2007). It accounts for 2.1% of skin infections occurring in the ICU (Gaynes and Edwards, 2005). In a group of victims with open tibial fractures, *Acinetobacter* was the most common agent found. Still, its pathogenicity appeared low, as it was not associated with any amputation when not detected in follow-up cultures (Johnson et al., 2007). In contrast, infections in burned military patients were more severe, leading to longer hospital stays and higher mortality rates (Albrecht et al., 2006). Osteomyelitis can also manifest as a complication of skin wound infections caused by *A. baumannii* (Davis et al., 2005). When *A. baumannii* infects a skin wound, it typically presents as cellulitis initially, appearing as a red, edematous lesion resembling peau d'orange. Subsequently, it may progress to a sandpaper-like lesion with multiple vesicles, which can evolve into hemorrhagic bullae (Sebeny et al., 2008). A fatal case study described a 41-year-old morbidly obese man with a history of alcoholic liver disease who developed cellulitis caused by MDR *A. baumannii*. Despite early antibiotic intervention, the infection progressed to extensive myositis and fat necrosis, requiring multiple surgical debridements (Ali et al., 2014). The patient ultimately succumbed to multiorgan failure despite aggressive treatment. This case underscores the challenges in managing MDR *A. baumannii* skin infections, emphasizing the importance of early recognition, appropriate antibiotic selection, and timely surgical intervention to improve patient outcomes.

### 4.6 Others

Rare manifestations of *A. baumannii* infections include endocarditis, often associated with prosthetic valve replacement (Menon et al., 2006; Olut and Erkek, 2005; Rizos et al., 2007; Starakis et al., 2006; Valero et al., 1999). Colonization of contact lenses by *A. baumannii* can lead to endophthalmitis, keratitis, corneal ulcers, and periorbital cellulitis, which may also occur following eye surgery or penetrating trauma. *Acinetobacter* ranks as the third most common cause of corneal ulcers, accounting for 7% of cases in one study (Corrigan et al., 2001; Wang et al., 1998; Mahajan, 1984; Mark and Gaynon, 1983; Miller, 2005; Mathews et al., 2005).

Patients undergoing mechanical ventilation in the ICU may develop sinusitis due to *Acinetobacter* infection, which can subsequently progress to pneumonia (Bert and Lambert-Zechovsky, 1996; Pneumatikos et al., 2006). Peritonitis can also occur during peritoneal dialysis, presenting symptoms such as abdominal pain and cloudy dialysate (Dandecha and Sangthawan, 2002; Galvao et al., 1989; Valdez et al., 1991).

## 5 Epidemiology

### 5.1 Global prevalence and distribution

*Acinetobacter spp*. are widespread organisms, with the first isolate, initially identified as *Micrococcus calcoaceticus* in 1911 by the Dutch microbiologist Beijerinck, originating from soil (Beijerinck, 1911). These highly ubiquitous organisms have diverse natural reservoirs that are found in nearly all environmental niches, ranging from water bodies, soils, crude oil, and solid surfaces to raw food products, wild animals, plants, humans, and even nectar (Al Atrouni et al., 2016b). In contrast, the natural habitats of *A. baumannii* are not well-defined, as it is primarily isolated from hospital environments, particularly in ICUs and communities with close contact (Antunes et al., 2014). Growing evidence suggests that a similar pattern is observed in veterinary medicine, with infection and colonization occurring in seriously ill animals in veterinary clinics and ICUs (Zordan et al., 2011). Numerous countries worldwide have reported the presence of *A. baumannii* in human body lice (La Scola and Raoult, 2004; Kempf et al., 2012a; Bouvresse et al., 2011). For instance, the highly antibiotic-susceptible *A. baumannii* strain SDF, which causes community-acquired infections, was initially isolated from the interior of body lice collected from homeless individuals in France (Fournier et al., 2006). Additionally, traces of *A. baumannii* have been identified in environmental soil and water body samples around the world (Ma and McClean, 2021). An early study found that over a third of soil samples from Hong Kong contained *Acinetobacter* spp., with *A. baumannii* making up 14.7% (*n* = 34; Houang et al., 2001). Later, Hrenovic et al. isolated an antibiotic-resistant *A. baumannii* strain from acid paleosol in Croatia (Hrenovic et al., 2014). *A. baumannii* colonization has also been noted in livestock, particularly in cattle and poultry. It has also been reported in horses, pigs, donkeys, mules, goats, rabbits, dogs, and cats, as well as in food products from these animals, with a considerable variability in prevalence among birds (Ma and McClean, 2021). *A. baumannii* is considered a nosocomial bacterium with high potential to exhibit MDR and virulence (Rice, 2008). The antimicrobial resistance of *A. baumannii* is of significant concern worldwide (World Health Organization, 2024). Clinical isolates of *A. baumannii* strains often show varying degrees of resistance to commonly prescribed antibiotics, including carbapenems, such as imipenem and meropenem. Being carbapenem resistant is considered a key marker for extensively resistant bacteria, as it signifies a broad range of co-resistance to unrelated classes of antibiotics (Tacconelli et al., 2018). Antimicrobial-resistant *A. baumannii* is found worldwide, across all six inhabited continents, but in varying percentages among countries. A study mapping the global prevalence of *A. baumannii* measures the percentage of carbapenem-resistant isolates among all clinically isolated *A. baumannii* strains documented over the past decade (Ma and McClean, 2021). A recent meta-analysis study reveals the widespread occurrence of CRAB across Asia (76.2% resistance rate) and the Americas (69.4 % resistance rate), where it has become a significant issue in the majority of the countries, with lower prevalence rates for Mongolia, Japan, and Canada (Boyd et al., 2019; Beig et al., 2024). This resistant strain is also widespread in South Africa, the Ivory Coast, and regions surrounding the Mediterranean, including Southern Europe, the Middle East, and North Africa (Ma and McClean, 2021). Carbapenem resistance was remarkably low in Ireland at just 4%, whereas the Philippines reported an alarmingly high rate of 96.1% (Beig et al., 2024). In Latin America, carbapenem resistance among *A. baumannii* is among the highest in the world, with rates exceeding 50% in many countries and reaching up to 90% in some regions (Chagas et al., 2024; Table 1). A multicountry cohort study in Oceania has found an alarmingly high prevalence of the carbapenem-resistant *A. baumannii* (CRAB) in Fiji and Samoa (Baleivanualala et al., 2023). The study also indicated the existence of an Oceania outbreak strain among hospitalized patients in New Zealand and Australia (Baleivanualala et al., 2023). Another systematic review in 2023 has shown a significant prevalence of the CRAB in the sub-Saharan Africa region (Arowolo et al., 2023). This leaves Canada, Mongolia, Japan, Western Europe, and the Nordic region with the lowest prevalence of CRAB so far (Table 1).

**Table 1:** Geographical variations in C = carbapenem-resistant *Acinetobacter baumannii* prevalence.

**Region**	**Carbapenem resistance (%)**	**Notable findings**	**References**
Asia–Pacific	<5% (Japan), 88% (South Korea), to 96.1% (Philippines).	Significant variation in resistance; carbapenem-hydrolyzing class-D β-lactamase genes like blaOXA-23-like are common.	Lee et al., 2023; Akeda, 2021; Lee et al., 2012a; Beig et al., 2024
Mexico	>60%	High proportion of CRAB infections in the neonatal population, a high prevalence of co-resistance to antibiotics, and a high rate of isolates carrying blaOXA-24 and blaIMP genes.	Castillo Bejarano et al., 2023
Latin America and the Caribbean	>50% up to 90%	High prevalence of HAP and VAP; resistance rates vary significantly between countries. In Latin America, carbapenem resistance in *A. baumannii* is predominant due to the oxacillinases OXA-23, OXA-58, and (in Brazil) OXA-143. With Brazil and Argentina having the highest percentages.	Chagas et al., 2024; Mohd Sazlly Lim et al., 2019; Rodríguez et al., 2018.
Eastern Asia	30% (Mongolia) to 50–97% (Nepal)	In Nepal, carbapenem-resistant (CR) *A. baumannii* and MDR-*A. baumannii* are highly prevalent with *bla*_OXA − 23_ gene. Another study from Nepal identified the primary CR gene within the ACB complex at one hospital was *bla*_OXA − 23 − like_, followed by *bla*_NDM − 1_. Lowest prevalence of hospital-acquired pneumonia and ventilator-associated pneumonia in Eastern Asia.	Beig et al., 2024; Gurung et al., 2024; Mohd Sazlly Lim et al., 2019; Joshi et al., 2017
North America	~50% (USA) to relatively low incidence (Canada)	CRAB isolates in the United States have an antimicrobial susceptibility profile defined as difficult-to-treat resistance. In Canadian hospitals, the occurrence of carbapenem-resistant *A. baumannii* (CRA) is low, with an incidence rate of 0.02 per 10,000 patient days and 0.015 per 1,000 admissions, showing no significant rise over time. Lower proportion (0.7%) of *A. baumannii* among all clinically isolated Gram-negative pathogens.	Bulens et al., 2024; Beig et al., 2024; Boyd et al., 2019; Lob et al., 2016
Middle East and North Africa	70–90%	Higher prevalence than North America; higher incidence (4.6%) of *A. baumannii* in hospital settings. The prevalence of carbapenemase-producing isolates in hospital settings ranged from 2.3 to 67.7% in North Africa.	Al-Rashed et al., 2023; Abouelfetouh et al., 2022; Lob et al., 2016; Manenzhe et al., 2015
Africa (sub-Saharan)	20–60%	Significant prevalence of carbapenem-resistant *A. baumannii*, particularly in hospital-acquired infections. The prevalence of carbapenemase-producing isolates in hospital settings ranged from 9 to 60% in sub-Saharan Africa.	Arowolo et al., 2023; Manenzhe et al., 2015
Europe	71–75% in Southern-Eastern Europe. 2.8% Northern Europe (Nordic) 6.3% Western Europe	Studies highlight the urgent need for comprehensive measures to combat the escalating threat of carbapenem-resistant *A. baumannii* infections in Eastern European countries. Carbapenem resistance was remarkably low in Ireland at just 4%.	Piotrowski et al., 2024; Beig et al., 2024; Ayobami et al., 2020
Oceania	8.5% (Australia) 77–100% (Fiji, Samoa)	All isolates from Fiji and Samoa harbored *bla*_OXA23_, *bla*_OXA66_, and *ampC_2_* gene, mediating resistance to β-lactam antimicrobials, including cephalosporins and carbapenems.	Baleivanualala et al., 2023; Lee et al., 2023; Ma and McClean, 2021
Conflict zones (Iraq, Afghanistan, Gaza, Ukraine)	Variable	Increased risk of infections due to weakened infection control in hospitals under strain.	Joly-Guillou, 2005; Moussally et al., 2023; Granata et al., 2024
Turkey (post-earthquake 2023)	Variable	Increased infections due to environmental stress and hospital strain.	Eryilmaz-Eren et al., 2024

This table presents the distribution of carbapenem-resistant *Acinetobacter baumannii* (CRAB) across different regions, highlighting significant variations in resistance rates, key findings, and associated genetic factors. Data indicate that resistance rates are highest in South Korea (88%), the Philippines (96.1%), and parts of Latin America and the Middle East (up to 90%), while regions, such as Japan (<5%), Canada, and Northern Europe, report significantly lower prevalence. Conflict zones and disaster-affected areas also show variable resistance patterns due to weakened infection control measures.

### 5.2 Geographical variations and prevalence of multidrug-resistant strains

The distribution of MDR *A. baumannii* varies across different regions worldwide due to varying selective environmental pressures, differences in hospital infection control practices, and the presence of endemic strains in certain hospitals. A study in the Asia–Pacific region has shown a significant geographic variation in the carbapenem resistance rates, ranging from <5% in Japan to 88% in South Korea (Lee et al., 2023). Carbapenem resistance in *A. baumannii* is primarily attributed to carbapenem-hydrolyzing class-D β-lactamase genes, including *bla*_*OXA*−51−*like*_*, bla*_*OXA*−23−*like*_*, bla*_*OXA*−24/40−*like*_*, and bla*_*OXA*−58−*like*_ (Lee et al., 2012a). The *bla*_*OXA*−23−*like*_*, bla*_*OXA*−24/40−*like*_, and *bla*_*OXA*−58−*like*_ genes are the three primary classes driving the global CR-AB epidemics (Hamidian and Nigro, 2019). Additionally, these gene classes exhibit different geographic distributions worldwide (Peleg et al., 2008). Geographical differences are also observed in hospital-acquired and community-acquired infections. For instance, Central America, Latin America, and the Caribbean have the highest prevalence of HAP and VAP caused by *A. baumannii*, whereas Eastern Asia has the lowest prevalence (Mohd Sazlly Lim et al., 2019). Although rare, the majority of the cases of CAP have been reported during the summer months in tropical and subtropical climates, highlighting the role of environmental factors in the geographic variation of infection (Falagas and Rafailidis, 2007).

Regional variations in the proportion of *A. baumannii* among all clinically isolated aerobic and facultative Gram-negative pathogens have been documented, with rates ranging from 0.7% in North America to 4.6% in the Middle East (Lob et al., 2016). Also, the prevalence of colonization of *A. baumannii* among livestock varies by the geographic location of the farms (Ma and McClean, 2021). In contrast, the connection between *A. baumannii* and infections following injuries in conflict zones, such as those in Iraq, Afghanistan, Gaza, and Ukraine (Joly-Guillou, 2005; Granata et al., 2024; Moussally et al., 2023) or after natural disasters like the 6 February 2023 earthquake in central and southeast Turkey (Eryilmaz-Eren et al., 2024) has been recently viewed as a possible phenomenon affecting the geographical distribution of the pathogen. There is some evidence suggesting that morphine use, which is common after battlefield or crush injuries, may exacerbate *A. baumannii* infections, potentially due to its immunosuppressive effects (Breslow et al., 2011). Table 1 summarizes the distribution of CARB worldwide.

Furthermore, a systematic review addressing *A. baumannii*'s regional heterogeneity and antimicrobial susceptibility reported higher antibiotic resistance rates in ICUs compared to non-ICU floors. However, MDR rates were high in both settings, especially in Europe and the Middle East (Lob et al., 2016). Similarly, MDR rates ranged from 77 to 87% in Africa, Asia, and Latin America, while they spanned 47% in North America and over 93% in the Middle East and Europe (Lob et al., 2016). Additionally, a recent 2021 review on the global prevalence of *A. baumannii* identified the Middle East as one of the heavily afflicted areas (Ma and McClean, 2021).

Despite its ubiquity, *A. baumannii* infections are not uniformly distributed worldwide; they vary geographically due to changing environmental conditions, differences in hospital infection control practices, and different strains across different countries.

### 5.3 Risk factors for infection

*A. baumannii* is a significant opportunistic pathogen responsible for healthcare-associated infections, particularly in hospitalized patients, including those in ICUs. It can cause a wide range of infections, such as VAP, bloodstream infections, urinary tract infections, and meningitis (Antunes et al., 2014). The established risk factors associated with *A. baumannii* infections include invasive procedures, prior hospitalization, host-specific factors, extended ICU stays, and previous broad-spectrum antibiotic use (Lin and Lan, 2014). Notably, patients with *A. baumannii* infections experience longer ICU stays and higher mortality rates, with the ICU mortality rate for VAP caused by MDR *A. baumannii* reaching up to 84.3% (Inchai et al., 2015). Mechanical ventilation is one of the primary contributors to the development of nosocomial pneumonia caused by MDR *A. baumannii*. A retrospective incidence study revealed that mechanical ventilation was associated with a 2.5-fold increased risk of acquiring nosocomial infections with MDR bacteria (Fang et al., 2016). Additionally, mechanical ventilation significantly increases the risk of developing healthcare-associated infections with MDR *A. baumannii*, further highlighting its role as a critical risk factor in ICU settings (Michalopoulos et al., 2011). Benaissa et al. performed a multivariate analysis, which concluded that mechanical ventilation, use of probabilistic antibiotic therapy for more than 4 days, prolonged invasive procedures, and neoplastic pathologies are independent risk factors for *A. baumannii* infection (Benaissa et al., 2023). The study also shows that prior exposure to carbapenem antibiotics, namely, imipenem and colistin, significantly increased the risk of *A. baumannii* infection by eighteen and 5 times, respectively (Benaissa et al., 2023). Also, several studies have identified prior exposure to antibiotics as a risk factor for MDR bacterial infections (Ang and Sun, 2018), highlighting the importance of rational use of antibiotics. Finally, host-specific factors such as underlying medical conditions or compromised immunity are regarded as risk factors for *A. baumannii* infection. For instance, neoplastic pathology increased the risk of *A. baumannii* infection by 5.7 times compared to the control group (Benaissa et al., 2023). Notably, during the COVID-19 pandemic, hospital-acquired co- or secondary infections caused by *A. baumannii* in COVID-19 patients were reported globally (Lai et al., 2020; Sharifipour et al., 2020). It has been reported that 85% of patients with hospital-acquired *A. baumannii* infections who developed VAP had co-infections with severe acute respiratory syndrome coronavirus 2 (SARS-CoV-2; Perez et al., 2020). In conclusion, patients admitted to intensive care units, those with indwelling medical devices, and individuals with immunocompromised conditions are particularly susceptible to *A. baumannii* infections.

### 5.4 Hospital-acquired vs. community-acquired infections

*A. baumannii* is commonly found in hospitals and often shows resistance. It has been isolated in intensive care units and detected on various reusable medical equipment, such as ventilators, humidifiers, blood pressure monitors, stethoscopes, laryngoscopes, curtains, mattresses, pillows, urinals, sinks, door handles, and even the gowns and gloves of hospital staff (Ahuatzin-Flores et al., 2024). Additionally, it has been found in bronchial and oropharyngeal secretions, as well as in the digestive tract of hospitalized patients (Almasaudi, 2018; Jung and Park, 2015). *A. baumannii* is a significant hospital pathogen, responsible for 2–10% of all Gram-negative HAIs (Ayobami et al., 2019; Fahy et al., 2023). In ICUs, it accounts for 20.9% of HAIs, with CRAB representing 13.6% of these infections (Ayobami et al., 2019). Hospitals across North America saw a sharp rise in *A. baumannii* resistance, escalating from 1.0% in 2003 to 58.0% in 2008. Meanwhile, the findings from the European Antimicrobial Resistance Surveillance Network (EARS-Net) reveal that carbapenem-resistant *Acinetobacter* species pose a significant public health risk in Europe. This issue is particularly severe in Southern and Eastern Europe, where over 70% of invasive isolates are resistant to carbapenems (Nguyen and Joshi, 2021). Its spread within healthcare premises is facilitated by its ability to survive in both dry and humid environments, with an ability to tolerate such conditions for long periods (Ahuatzin-Flores et al., 2024). Moreover, *A. baumannii's* resistance to disinfectants and antibiotics, combined with its ability to form biofilms, allows it to colonize inert surfaces and medical devices. This characteristic is ascribed to the presence of capsular polysaccharides, enabling *A. baumannii* to survive in nutrient-limiting conditions (Ramirez et al., 2020; Anane et al., 2019). Community-acquired *A. baumannii* infections are rarely reported, but they are more common in certain tropical or subtropical regions, suggesting that the pathogen may be acquired from sources outside of hospitals (Ahuatzin-Flores et al., 2024). The presence of *A. baumannii* in out-of-hospital environments remains debatable. There is a lack of definitive evidence concerning its natural habitat beyond hospitals, how it is introduced into hospital settings, and its potential re-emergence in the environment (Ahuatzin-Flores et al., 2024). Nonetheless, several studies have identified *A. baumannii* in multiple non-hospital settings, including aquatic environments (Galarde-López et al., 2022), livestock (Ghaffoori Kanaan et al., 2020), soil (Suresh et al., 2022), food (Bitar et al., 2019; Cho et al., 2018), produce (Berlau et al., 1999; Malta et al., 2020), domestic pets (Kimura et al., 2018; Lysitsas et al., 2023; Wareth et al., 2019), wildlife (Deng et al., 2021), and even human body lice (Ly et al., 2020). In contrast to healthcare-associated infections, *A. baumannii* strains responsible for community-acquired infections tend to be more susceptible to antibiotics. Farrugia et al. sequenced the entire genome of a community strain, D1279779, and discovered that it lacked the antibiotic resistance island AbaR, which is commonly found in nosocomial strains (Farrugia et al., 2013). Although community-acquired *A. baumannii* infections are relatively rare, they often present with a severe and rapidly progressive course, including septic shock, respiratory failure, severe sepsis, and pneumonia, with a considerably high mortality rate (Fang et al., 2016; Farrugia et al., 2013; Brigo et al., 2022).

## 6 Antibiotic resistance

Among the six pathogens contributing most significantly to the burden of deaths due to antimicrobial resistance in 2019, CRAB emerged as the fourth most burdensome globally (Antimicrobial Resistance Collaborators, 2022). *A. baumannii* is classified among the MDR ESKAPE organisms (*Enterococcus faecium*, Staphylococcus aureus, *Klebsiella pneumoniae, A. baumannii, P. aeruginosa*, and *Enterobacter* species), recognized as critical priority pathogens by the WHO. These pathogens, designated as Priority 1 tiers, have developed resistance to a wide range of first-line and last-resort antibiotics, including carbapenems and third-generation cephalosporins (WHO, 2017). Consequently, patients infected with carbapenem-resistant pathogens, including *A. baumannii*, face heightened risks of morbidity and mortality, with bloodstream infections contributing to poor outcomes and limited therapeutic options (van Duin et al., 2013). Furthermore, the widespread dissemination of MDR *A. baumannii* strains has been exacerbated by the overuse of antibiotics and inadequate antibiotic usage (van Duin et al., 2013; Endale et al., 2023). Consequently, *A. baumannii* has been classified based on its antibiotic sensitivity into MDR, XDR, and pan-drug-resistant (PDR) phenotypes (Vrancianu et al., 2020).

### 6.1 Mechanisms of resistance to antibiotics

To counteract the effects of antibiotics, *A. baumannii* employs a diverse array of antibiotic resistance mechanisms, each targeting specific types of drugs. These mechanisms include enzymatic degradation, reduced membrane permeability, increased efflux mechanisms, target site modification, and genetic mechanisms of resistance (Vrancianu et al., 2020; Ibrahim et al., 2021).

#### 6.1.1 Enzymatic warfare

*A. baumannii* can evade the effects of some antibiotics by producing certain enzymes. Consequently, the bacteria can hydrolyze antibiotics or modify the antibiotic's structure by transferring functional groups (Blair et al., 2015). An example of these enzymes is the β-lactamases, among the oldest known enzymes that cleave the β-lactam ring structure (Pollock, 1967). β-Lactam antibiotics, which include penicillins, cephalosporins, carbapenems, monobactams, and β-lactamase inhibitors, target the penicillin-binding proteins (PBPs) of bacteria (Pandey and Cascella, 2024). The key feature of β-lactamase enzymes is their ability to render ineffective a broad range of β-lactams, including some of the most potent carbapenems and extended-spectrum cephalosporins, thus augmenting and amplifying *A. baumannii*'s resistance to β-lactam antibiotics (Aurilio et al., 2022). Furthermore, enzymes can inactivate antibiotics by transferring chemical groups onto the antibiotic molecules, preventing them from binding to their target proteins. For instance, *A. baumannii* can produce aminoglycoside-modifying enzymes (AMEs) such as acetyltransferases, nucleotidyltransferases, or phosphotransferases, leading to enzymatic modification of aminoglycoside antibiotics. Consequently, aminoglycosides will no longer be able to exert their effects on the bacteria. Through mutagenesis and the transferability of encoding genes at the molecular level, AMEs enable the acquisition of heightened aminoglycoside resistance (Ramirez and Tolmasky, 2010).

#### 6.1.2 Reduced membrane permeability

Gram-negative bacteria possess a complex outer membrane composed of phospholipids, lipopolysaccharides, lipoproteins, and β-barrel porins, forming a permeability barrier. This intrinsic complexity renders them less permeable to many antibiotics through the regulation of porins or substitution with more-selective channels (Blair et al., 2015; Choi and Lee, 2019; Maher and Hassan, 2023). Previously, limited knowledge about *A. baumannii* included sparse data on its outer membrane proteins and permeability (Vila et al., 2007). However, the primary and most abundant OM protein, outer membrane protein A (OmpAb), a 40 kDa porin, plays a crucial role in reducing membrane permeability (Jyothisri et al., 1999). Further studies, including one by Nikaido and Sugawara, confirmed that OmpAb forms a low permeability channel, serving as the “principal non-specific slow porin” of *A. baumannii* (Sugawara and Nikaido, 2012).

#### 6.1.3 Increased efflux mechanisms

Efflux mechanisms represent a fundamental strategy bacteria employ to develop resistance to drugs, including antibiotics. These cell-membrane-lodged efflux pumps enable bacteria to regulate their inner environment by transporting molecules, such as antibiotics, outside of the cell (Pearson et al., 1999). As previously noted, several principal categories of efflux pumps have been identified in *A. baumannii*: MFS, RND, SMR, MATE, ABC, and PACE (Lin et al., 2014), with MFS, RND, SMR, and MATE families being the most frequently correlated to resistance.

MFS efflux pumps, including Tet(A) and Tet(B), confer resistance to tetracyclines in *A. baumannii* (Martí et al., 2006). Recently, Gaona et al. identified a novel MFS efflux pump SxtP as well as its associated activator **L**ysR-**t**ype **t**ranscriptional **r**egulator (LTTR) SxtR, both of which are involved in mediating resistance to sulfamethoxazole/trimethoprim (Gaona et al., 2024).

The AdeABC pump, belonging to the RND superfamily, was the first pump identified in *A. baumannii* and is associated with decreased susceptibility to various antibiotics, including aminoglycosides, tetracycline, erythromycin, chloramphenicol, fluoroquinolones, and β-lactams (Fournier et al., 2006; Vila et al., 2007; Magnet et al., 2001). In a recent study tackling the resistance role of RND efflux pumps, *A. baumannii* isolates recovered from tertiary care hospitals of three western Balkan countries revealed that the overexpression of RND efflux pumps mediates resistance to tigecycline. This further accentuates the significant role of such pumps in antibiotic resistance (Novović et al., 2025).

AbeS, a central efflux pump of the SMR family, is widespread in MDR strains of *A. baumannii*, contributing to antibiotic resistance (Srinivasan et al., 2009). MATE efflux pumps represent another family of pumps that function as antiporters by pumping out cations for hydrogen or sodium ions. For instance, the AbeM efflux pump belonging to MATE has proved to have increased resistance to norfloxacin, ciprofloxacin, and ofloxacin (Zack et al., 2024). Additionally, the A1S_3371 MATE efflux pump enhances the virulence of *A. baumannii* (Pérez-Varela et al., 2019).

The ATP-binding cassettes, such as the MacAB-TolC system, are overexpressed in mature biofilms of *A. baumannii*, aiding in adaptation to deleterious conditions (Robin et al., 2021). Furthermore, the PACE family proteins, such as Acel, assist in resisting chlorhexidine, a widely used antiseptic (Hassan et al., 2015).

In summary, *A. baumannii* employs various efflux mechanisms to transport drugs out of the cell, reducing drug concentration inside and compromising treatment efficacy.

#### 6.1.4 Target site modification

When administered, antibiotics usually bind tightly to target sites within bacteria, halting bacterial growth and activity. However, once these target sites undergo changes, the antibiotic loses its capacity to bind effectively, leading to resistance (Blair et al., 2015). One method by which resistance is conferred is via transformation, which involves the uptake of DNA from the environment to form mosaic genes, enabling modification of target proteins (Blair et al., 2015). *A. baumannii* can develop resistance to various antibiotics through such target site modifications, either by genetic mutations in specific sites or by post-transcriptional modifications in diverse proteins (Kyriakidis et al., 2021; Singh et al., 2013). Target site modification fosters resistance against a plentiful array of antimicrobials, such as β-lactams, macrolides, fluoroquinolones, aminoglycosides, including polymyxin resistance, namely, colistin (Martínez-Trejo et al., 2022). For instance, imipenem, a carbapenem antibiotic, inhibits the activity of its target site, penicillin-binding protein 2 (PBP2). Over time, *A. baumannii* has developed molecular adaptations by reducing the affinity of PBP2 for Imipenem, enabling it to resist the effects of this antibiotic (Gehrlein et al., 1991). Similarly, *A. baumannii* exhibits fluoroquinolone resistance through mutations in genes such as *gyrA, gyrB*, and *parC*, which encode type-II and type-IV topoisomerases, the targets of fluoroquinolones (Vrancianu et al., 2020; Redgrave et al., 2014). In addition, for the antibiotic Linezolid, mutations by base substitutions in the V domain of the 23S rRNA and the presence of a transmissible Cfr(B) rRNA 23S methyltransferase can occur as a target site modification, while being the most prevalent mechanism of resistance of A. baumannii to this antibiotic (Martínez-Trejo et al., 2022). Colistin has long been used for MDR A. baumannii, and resistance to colistin has been described in several *in vitro* and *in vivo* studies. In *A. baumannii*, this mechanism is governed by the mutation of the *pmrA* and/or *prmB* protein genes along with the constitutive expression of *prmA*, leading to the positive regulation of the *pmrCAB* operon and the addition of phosphoethanolamine to the phosphate of LPSs (Martínez-Trejo et al., 2022).

#### 6.1.5 Genetic mechanisms of resistance

Resistance can be acquired by emerging mobile genetic elements (MGEs) in *A. baumannii*. Transposons, plasmids, and integrons facilitate the spread of MDR strains by enabling the bacteria to acquire new resistance genes or transfer existing ones to other bacteria (Ibrahim et al., 2021).

Transposons facilitate antibiotic resistance in *A. baumannii* through various mechanisms, including enhanced expression of resistance genes by potent promoter sequences, disruption of expression due to promoter disruption by transposons, and direct gene activation through insertion within coding sequences (Noel et al., 2022). For instance, insertion sequence (IS) elements promote *A. baumannii* clinical resistance to carbapenems by furnishing strong promoter sequences to silence *bla* genes encoding β-lactamase enzymes (Noel et al., 2022). By downregulating gene expression, IS can also confer resistance. A study done by Palmer et al. identified a suppressor mutation (IS*Aba11* transposition) that reduced the expression of the isoprenoid biosynthesis gene *ispB*, thus establishing resistance to different antibiotics, including meropenem, imipenem, and gentamicin (Palmer et al., 2020). Plasmids are another mobile genetic element utilized by *A. baumannii* to acquire resistance. They can be transferred between bacteria either vertically or horizontally. Vertically transmitted plasmids are those disseminated from a parent cell to the progeny, while horizontally transmitted plasmids are conveyed by conjugation (Partridge et al., 2018). For example, XerCD-dif site-specific recombination systems are flanked by antibiotic-resistance genes on plasmids, creating a potential mechanism for acquiring and disseminating resistance genes (Balalovski and Grainge, 2020). To understand how different types of plasmids contribute to the spread of AMR, another recent study conducted by Lam and Hamidian analyzed the distribution of varying plasmid types and antimicrobial resistance genes on *A. baumannii* plasmids. They showed that many RP-T1 and R3-T2 types, and *rep*-less pNDM plasmids can spread various carbapenem resistance genes, noting that carbapenems are often among the last-resort antibiotics for MDR *A. baumannii* (Lam and Hamidian, 2024).

Integrons, which *Acinetobacter* species harbor, are aligned with antibiotic resistance acquisition and serve as a significant source of resistance genes in Gram-negative bacteria, including *A. baumannii* (Nemec et al., 2004). High prevalence of integron class 1 in *A. baumannii* isolates has been observed, contributing to resistance to a wide range of antibiotics through the transmission of resistance genes (Ghazalibina et al., 2019). Another study by Shetty et al. identified the presence of class-1 integrons in *A. baumannii* and detected new variants of resistance genes within the strain AB34: *AAC(6*′*)-Ib-CatB8-ANT(3*″*)-Ia-AadA1*, which code for amikacin, chloramphenicol, and streptomycin. In addition, integrons of the strains AB36 and AB41 contained a rare gene cassette *CmlA5-Arr2* coding for chloramphenicol and rifampin (Shetty et al., 2024). In summary, *A. baumannii* can evade antibiotics by acquiring and disseminating various resistance genes in its genome through mobile genetic elements.

### 6.2 Impact of antibiotic resistance of *A. baumannii* on clinical outcomes and treatment options

Bacteria resistant to antibiotics render these drugs ineffective, making infections difficult to treat and leading to a notable escalation in mortality rates and deterioration in clinical outcomes (WHO, 2023). Compared to non-infected patients and those infected with susceptible strains of *A. baumannii*, infections with MDR *A. baumannii* are associated with prolonged hospital and ICU stays and more unfavorable clinical outcomes (Sunenshine et al., 2007). Among hospitalized patients with MDR *A. baumannii* bacteremia, higher mortality rates and medical financial burden and costs have been observed compared to non-MDR *A. baumannii* bacteremia. Remarkably, MDR *A. baumannii* bacteremia resulted in 13.4 days of supplementary hospitalization and US$3,758 of additional costs (Lee et al., 2007).

During the COVID-19 pandemic, patients affected by both MDR *A. baumannii* and COVID-19 had an augmented mortality risk, illustrating the pronounced impact of simultaneous infections with MDR *A. baumannii* on clinical outcomes (Alenazi et al., 2023). Additionally, a recent Lebanese study demonstrated the perturbing mortality rate of *A. baumannii* infections, especially in patients where improper initial antimicrobial treatment was initiated with existing antimicrobial resistance (Itani et al., 2023). Another “16-year retrospective cohort study” displayed the characteristics of carbapenem-resistant Gram-negative bacterial meningitis associated with *A. baumannii* and concluded that it is associated with prominent in-hospital mortality rates (Xu et al., 2024).

Regarding the impact of *A. baumannii* antibiotic resistance on treatment options, numerous studies have shown an increasing rate of resistance against carbapenems and colistin, especially in Asia, Latin America, Europe, and Australia (Kumar et al., 2021). Over time, *A. baumannii* has developed resistance to colistin via two main mechanisms: the complete loss of the target lipopolysaccharide or its modification (Novović and Jovčić, 2023). As a result, combined therapy with synergic drugs is endorsed when all conventional treatments become ineffective against MDR *A. baumannii* and may even be the exclusive therapeutic option for PDR *A. baumannii* (Kumar et al., 2021; Karakonstantis et al., 2021).

### 6.3 Current drugs with resistance

Before the 1970s, ampicillin, carbenicillin, gentamicin, and nalidixic acid, whether given alone or in combination therapy, were considered optimal for treating *Acinetobacter* infections. However, after 1975, resistance levels soared significantly (Manchanda et al., 2010). Consequently, *A. baumannii* has developed resistance to several current drugs.

#### 6.3.1 β-lactams

As previously discussed, β-lactam antibiotics (penicillins, cephalosporins, carbapenems, monobactams, and β-lactamase inhibitors) share a common β-lactam ring structure and target bacterial PBPs (Pandey and Cascella, 2024). *A. baumannii* has developed resistance to β-lactam antibiotics through various mechanisms, including hydrolysis by β-lactamases, increased expression and activity of efflux pumps, decreased membrane permeability leading to reduced influx, and modification of target sites (Kyriakidis et al., 2021). In Gram-negative bacteria like *A. baumannii*, the primary mechanism of β-lactam resistance is the synthesis of β-lactamase enzymes (Matthew and Harris, 1976).

β-Lactamases are classified according to the Ambler classification, which groups these enzymes into four classes (A, B, C, and D) based on molecular analysis and amino acid sequences. Classes A, C, and D enzymes utilize a serine amino acid for β-lactam hydrolysis, forming an acyl enzyme intermediate, while class-B β-lactamases are zinc-dependent metalloenzymes (metallo-β-lactamases, MBLs) that require at least one active-site zinc ion for substrate hydrolysis (Ambler, 1980; Bush and Jacoby, 2010).

According to the findings summarized in Kyriakidis et al.'s review, Class A β-lactamases, including SHV, CTX M, and KPC, confer resistance to penicillins, cephalosporins, monobactams, and carbapenems (Kyriakidis et al., 2021; Tooke et al., 2019). Catalysis by class-B β-lactamases (VIM, IMP, SPM, GIM, and NDM) extends to the breakdown and hydrolysis of almost all β-lactams, except monobactams (Kyriakidis et al., 2021; Kateete et al., 2016).

Ambler class-C β-lactamases, also known as chromosomally encoded *Acinetobacter*-derived cephalosporinases (ADCs), contribute to resistance against cephalosporin antibiotics through an ISAba1 insertion sequence close to the resistance genes (Kyriakidis et al., 2021).

Class-D β-lactamases, also known as oxicillinases (OXA) or carbapenem-hydrolyzing class D-lactamases (CHDLs), particularly those of the OXA-10 family, can render all β-lactam antibiotics inactive, including carbapenems (Kyriakidis et al., 2021). Over 400 OXA enzymes have been identified, with the largest being the OXA-51-like β-lactamases identified to date (Monem et al., 2020; Evans and Amyes, 2014).

Resistance to carbapenems often depends on β-lactamases of classes B (MBLs) and D (OXA-type carbapenemases; Ibrahim, 2019). For instance, the OXA-23 enzyme, one of the Ambler class-D β-lactamases, is sufficient to confer resistance to carbapenem antibiotics (Evans and Amyes, 2014).

The other classification of β-lactamases, proposed by Bush, Jacoby, and Medeiros, was based on the enzymes' ability to cleave specific β-lactam classes and their susceptibility to β-lactam inhibitors (Bush and Jacoby, 2010; Bush et al., 1995). According to this classification, β-lactamases were grouped into three categories: group-1 cephalosporinases, group-2 serine β-lactamases, and group-3 metallo-β-lactamases (Bush and Jacoby, 2010).

In ICUs, CRAB is emerging as a major cause of healthcare-associated infections (Tomczyk et al., 2019). Designated by the WHO as a critical-priority bacterium, CRAB ranks among the top 12 pathogens exhibiting resistance, posing significant public health challenges and necessitating urgent action (Tacconelli et al., 2018). One subset of CRAB, known as *carbapenemase* gene-positive CRAB (CP-CRAB), carries antibiotic resistance genes encoded on mobile genetic elements and produces carbapenemase enzymes. These enzymes confer remarkable resistance by degrading the majority of the β-lactam antibiotics, including carbapenems, making CP-CRAB infections particularly concerning.

In a recent scientific review on carbapenem resistance in *A. baumannii*, Nguyen and Joshi summarized the mechanisms into four groups: alterations in penicillin-binding proteins, membrane impermeability due to loss of OMPs, overexpression of efflux pumps, and carbapenem inactivation through synthesis of carbapenemases (Nguyen and Joshi, 2021).

#### 6.3.2 Fluoroquinolones

Fluoroquinolones, derivatives of quinolones with added fluorine atoms, belong to the family of broad-spectrum antibiotics effective against a wide range of aerobic Gram-positive and Gram-negative organisms. These antibiotics disrupt bacterial mRNA synthesis and DNA replication by inhibiting type-II and type-IV DNA topoisomerases. Common fluoroquinolone drugs include ciprofloxacin, Gemifloxacin, Levofloxacin, Moxifloxacin, Norfloxacin, and Ofloxacin (National Institute of Diabetes and Digestive and Kidney Diseases, 2012).

*A. baumannii* commonly develops resistance to fluoroquinolones through various mechanisms, with target site mutation being the most prevalent. Mutations in the *gyrA, gyrB*, and *parC* genes, which encode gyrase and topoisomerase IV, allow bacteria to acquire resistance. For example, clinical isolates of *A. baumannii* from Mansoura University hospitals in Egypt showed resistance to ciprofloxacin primarily through *gyrA* and *parC* mutations, with combined mutations accounting for 85.5% of cases (Zaki et al., 2018). Similarly, studies have demonstrated a strong association between fluoroquinolone resistance and mutations in the *gyrA, gyrB*, and *parC* genes (Park et al., 2011). Recent research using EUCAST antimicrobial susceptibility testing has highlighted the increasing resistance of *A. baumannii* to ciprofloxacin, surpassing EUCAST breakpoints and reducing its efficacy against the bacterium (Darby et al., 2024).

In addition to target site mutations, fluoroquinolone resistance in *A. baumannii* can also occur through plasmidic and chromosomal efflux mechanisms, reduced membrane permeability, and transmissible mechanisms mediated by plasmids (plasmid-mediated quinolone resistance genes; Redgrave et al., 2014).

#### 6.3.3 Aminoglycosides

Aminoglycosides, such as gentamicin, amikacin, tobramycin, neomycin, and streptomycin, are broad-spectrum bactericidal antibiotics that inhibit protein synthesis in Gram-negative pathogens (Germovsek et al., 2017; Brewer, 1977; Krause et al., 2016).

In *A. baumannii*, resistance to aminoglycosides can occur through three distinct mechanisms, with the most prevalent being the alteration of hydroxyl or amino groups of these antibiotics by AMEs, which reduces their binding capacity. Other mechanisms include efflux mechanisms and target site modification (ribosomal binding protein; Kyriakidis et al., 2021; Asif et al., 2018).

AMEs, such as acetyltransferases, nucleotidyltranferases, or phosphotransferases, enhance *A. baumannii*'s ability to develop aminoglycoside resistance through gene transfer and mutagenesis (Ramirez and Tolmasky, 2010). For example, a 2019 study analyzing 1,200 bacterial isolates from ICU patients found high levels of resistance, with various types of aminoglycoside-modifying genes detected in *A. baumannii* isolates, including aadB, aphA6, aadA1, and aacC1 (Rizk and Abou El-Khier, 2019).

Efflux mechanisms also contribute significantly to aminoglycoside resistance in *A. baumannii*. Research has identified seven gene products in *A. baumannii* involved in successful aminoglycoside removal through efflux mechanisms, including two-component systems, pumps, permeases, and periplasmic adaptors (Kyriakidis et al., 2021; De Silva and Kumar, 2019).

Finally, emerging resistance of *A. baumannii* to clinically useful aminoglycosides, such as gentamicin, tobramycin, and amikacin, can occur through target site modification via methylation of 16S ribosomal RNA (Doi and Arakawa, 2007).

#### 6.3.4 Tetracyclines

Tetracyclines, including doxycycline, minocycline, and tigecycline, are broad-spectrum antibiotics that inhibit bacterial growth by targeting the 30S ribosomal subunit, thereby preventing protein synthesis (Shutter and Akhondi, 2024).

In *A. baumannii*, resistance to tetracyclines is achieved through various mechanisms, including ATP-driven efflux mechanisms mediated by RND pumps (AdeABC and AdeIJK) and MFS pumps (TetA and TetB), drug enzymatic inactivation, and ribosomal protection proteins (RPPs), such as TetM, TetW, TetO, and TetSf (Kyriakidis et al., 2021; Warburton et al., 2016).

Recent evidence suggests promising developments in overcoming tetracycline resistance with aminomethylcyclines KBP-7072 and omadacycline, third-generation tetracyclines. These compounds have demonstrated the ability to circumvent efflux and RPPs tetracycline resistance mechanisms *in vitro*, offering potential avenues for the development of “anti-resistant” tetracycline-based antibiotics (Huband et al., 2020).

However, the effectiveness of tigecycline against *A. baumannii* has been compromised by the emergence of plasmid-mediated *tet(X)* mono-oxygenase gene variants. These bacterial mono-oxygenases have the capacity to render all tetracyclines, including tigecycline, eravacycline, and omadacycline, inactive (Kyriakidis et al., 2021). He et al. found that *tet*(X3) and *tet*(X4) increased the tigecycline minimal inhibitory concentration values for *E. coli, K. pneumoniae*, and *A. baumannii* and notably compromised tigecycline in *in vivo* infection models (He et al., 2019). Consequently, a novel, plasmid-mediated tigecycline resistance *tet*(X) gene variant, *tet*(X5), was detected in a clinical *A. baumannii* isolate from China in 2017. Whole-genome sequencing confirmed the sequence of *tet*(X) from AB17H194, where it conferred resistance to tigecycline, eravacycline, and omadacycline (Wang et al., 2019).

#### 6.3.5 Polymyxins

Polymyxins, comprising polymyxin B and polymyxin E (colistin), serve as antibiotics for treating Gram-negative MDR infections, including those caused by *A. baumannii*. They function by altering and destabilizing the LPS of the outer bacterial membrane (Shatri and Tadi, 2024).

As polymyxins, particularly colistin, emerge as the last line of defense against MDR *A. baumannii* infections, the rise of colistin-resistant *A. baumannii* strains presents significant public health implications. The escalating prevalence of these strains, particularly in healthcare settings, can be attributed to selective pressure on bacteria and the extensive use of polymyxins, even against carbapenem-resistant Gram-negative bacteria (Lima et al., 2020).

*A. baumannii*'s resistance to polymyxins can occur through two primary mechanistic modalities: the complete loss or modification of the target LPS. This resistance can be chromosomally or plasmid mediated. Mechanisms include mutations in genes responsible for synthesizing lipid-A and LPS cofactors, alterations in the outer membrane structure through modifications of lipid A, metabolic shifts affecting amino acids, and the upregulation of efflux pumps (Lima et al., 2018).

#### 6.3.6 Combination therapy

While combination therapies are often employed to combat MDR*A. baumannii* infections, it is important to note that resistance mechanisms can also develop against these combinations. A review of 12 studies involving 1,040 patients with MDR *Acinetobacter* infections reported variable mortality rates, ranging from 27 to 57.1% in the combination therapy groups. Although some combinations, such as carbapenem with colistin and carbapenem with ampicillin/sulbactam, showed improved outcomes, the benefit was inconsistent across all studies. Furthermore, resistance emergence was observed with off-label tigecycline-based regimens, underscoring the adaptability of *A. baumannii* to combination treatments (Poulikakos et al., 2014). Additionally, a systematic review of 84 studies evaluating 818 *A. baumannii* isolates resistant to all components of combination therapy found that while specific double and triple polymyxin-based regimens exhibited synergy at clinically relevant concentrations, no combination was universally effective against all strains. Some combinations showed synergy only at concentrations unlikely to be clinically achievable (Karakonstantis et al., 2021).

This adaptation poses challenges in combating *A. baumannii* infections and underscores the need for effective antimicrobial stewardship to preserve the efficacy of polymyxins in clinical settings.

## 7 Diagnostic approaches

Rapid and accurate identification of *A. baumannii* is clinically crucial, as it enables timely and precise diagnosis, which has significant therapeutic implications, enabling healthcare providers to implement targeted and effective treatment strategies for managing severe infections caused by this bacterium. For the diagnosis of *A. baumannii*, many tests can be used, either phenotypic or molecular, and they are used primarily nowadays (Tables 2, 3).

**Table 2:** Diagnostic methods for *Acinetobacter baumannii*.

**Method type**	**Diagnostic technique**	**Key features**
Phenotypic	Blood, chromogenic, MacConkey agar culture	Identifies bacterial growth using Gram stain and colony morphology.
	AntibioGram (disk diffusion)	Measures resistance by antibiotic zone inhibition for various antibiotics.
	Biofilm detection	Detects biofilm, a virulence factor increasing resistance, via polystyrene blue assay and crystal violet staining.
Molecular	RAPD	Confirms *A. baumannii* and detects resistance genes like carbapenemase.
	Multiplex PCR/multiplex RT-PCR	Detects multiple resistance genes simultaneously.
	PCR-ESI/MS	Identifies silent mutations and genetic rearrangements.
	Repetitive sequence-based PCR	Detects repetitive sequences in the bacterial genome.
	MLVA	Detects tandem repeats for strain differentiation.
	PFGE	Gold standard for studying isolated clonality in epidemiology.
	AFLP	Characterizes strains by single-locus (e.g., *adeB* and *rpoB*) and multilocus sequencing (e.g., trilocus sequencing).
	LAMP	Rapid, cost-effective detection of carbapenemase genes (*ISAba1-blaOXA-51, recA*, and *OXA-23*).
	Multiple LAMP	Allows simultaneous detection of multiple genes.
	LOAD	Combines DNA extraction, LAMP amplification, and detection; highly specific, rapid for critically ill patients.

*A. baumannii, Acinetobacter baumannii*; PCR, polymerase chain reaction; RT-PCR, reverse transcription polymerase chain reaction, RAPD, random amplified polymorphic DNA, PCR-ESI/MS, PCR-electrospray ionization mass spectrometry; MLVA, multiple locus variable number tandem repeat analysis; PFGE, pulse field gel electrophoresis; AFLP, amplified fragment length polymorphism; LAMP, loop-mediated isothermal amplification; LOAD, lab-on-a-disk.

**Table 3:** Comparison of the different diagnostic methods for *Acinetobacter baumannii*.

**Method**	**Sensitivity**	**Specificity**	**Turnaround time**	**Cost**	**Clinical applicability**
Traditional culture	Moderate (species-level ambiguity)	High (98–100%)	24–72 h	Low (US$5–20)	Limited-resource settings
Absorbance assay	97.6%	99.6%	90 min	Low (US$10–15)	High-throughput labs with limited resources
MALDI-TOF MS	98%	100%	2.5–4 h	High (US$50–100)	High-tech hospitals
CarbAcineto NP test	94.7%	100%	2 h	Low (US$10–15)	Resource-limited labs
CarbAcineto NP + sCIM algorithm	98.18%	100%	~3–4 h	Low (US$15–20)	Optimized for low-resource settings
mCIM	32.73%	Not reported	8–24 h	Low (US$5–10)	Limited utility due to low sensitivity
PCR	98.9% (species-specific)	100% (species-specific)	1–4 h	Moderate (US$20–50)	High-throughput labs, species identification
PFGE (pulsed-field gel electrophoresis)	High (for typing)	High (for typing)	24–48 h	High (US$50–100)	Molecular epidemiology, outbreak tracing
AFLP (Amplified Fragment Length Polymorphism)	High (for typing)	High (for typing)	24–48 h	High (US$50–100)	Molecular epidemiology, strain differentiation
LAMP (Loop-mediated isothermal amplification)	100%	100%	30 min−1 h	Low (US$10–20)	Rapid point-of-care diagnostics, resource-limited settings
LOAD (Not commonly used for *A. baumannii*)	N/A	N/A	N/A	N/A	N/A

MALDI-TOF MS, matrix-assisted laser desorption–ionization time-of-flight mass spectrometry; PCR, polymerase chain reaction; MLVA, multiple locus variable number tandem repeat analysis; PFGE, pulse field gel electrophoresis; AFLP, amplified fragment length polymorphism; LAMP, loop-mediated isothermal amplification; LOAD, lab-on-a-disk.

### 7.1 Laboratory methods

#### 7.1.1 Traditional and automated phenotypic methods

In cases where *A. baumannii* is suspected, a specimen is typically obtained and cultured on blood agar, chromogenic agar, and MacConkey agar (Thermo Fisher Scientific, Basingstoke, UK). These cultural methods aid in identifying bacterial growth based on Gram stain and colony morphology (Khan et al., 2015). These traditional methods are commonly used in clinical settings, although they are time-consuming and may have limited sensitivity for detecting *A. baumannii* in low-density infections (Ayoub Moubareck and Hammoudi Halat, 2020).

Additionally, the antibiogram, an antibiotic sensitivity test, offers another means of detecting *A. baumannii*, primarily utilizing the disk diffusion method. This method, outlined in the Clinical and Laboratory Standards Institute (CLSI) guidelines, specifically measures resistance to colistin using the agar dilution method (Performance, 2016). It involves impregnating bacteria with specific antibiotics and measuring the diameter of the antibiotic zone of inhibition. This method is widely used but can be slow and limited in its ability to detect all resistance mechanisms. Adewoyin et al. reported that *A. baumannii* demonstrates resistance to various antibiotics, including piperacillin-tazobactam (11.2%), ceftazidime (12%), cefotaxime (18.8%), cefepime (8.8%), imipenem (2.7%), meropenem (4.15%), amikacin (2.4%), gentamicin (8.8%), tetracycline (16.8%), ciprofloxacin (11%), and trimethoprim/sulfamethoxazole (20.5%). Furthermore, this bacterium is known for its MDR (Adewoyin et al., 2021). matrix-assisted laser desorption–ionization time-of-flight mass spectrometry (MALDI-TOF MS) is able to identify *A. baumanii* with 100% specificity when combined with chemometric tools. It detects β-lactamase activity by analyzing antibiotic hydrolysis (e.g., imipenem degradation by carbapenemases), Nonetheless, it is limited to β-lactamase-mediated resistance; efflux pumps or porin mutations require supplementary testing. This method is beneficial because of its ability to directly detecting resistance in 2.5–4 h from blood cultures. MALDI-TOF MS is highly costly, but it reduces long-term labor and consumable costs (Bagudo et al., 2020).

Moreover, MCDA-LFP can simultaneously identify *A. baumannii* (via *pgaD*) and carbapenem resistance (via *bla_OXA-23-like*) with 100% specificity and in <1.5 h. To note that it is a cost-effective method as it eliminates the need for thermocyclers, reducing infrastructure costs (Hu et al., 2019).

CarbAcineto NP test is beneficial as it detects all carbapenemase types (OXA, GES, metallo-β-lactamases) with 94.7% sensitivity and 100% specificity, by outperforming traditional biochemical assays, which fail to detect non-MBL carbapenemases. This method is cost-effective as it uses affordable reagents, gives rapid results (in 2 h), and requires minimal training, rendering it suitable for resource-limited settings (Dortet et al., 2014).

A notable phenotypic characteristic of *A. baumannii* is its ability to form biofilms, which are considered virulence factors enabling the bacteria to persist on both biotic and abiotic surfaces (Khalawetektook and Tektook, 2018; Goodman, 1988). Biofilm formation also contributes to increased resistance to antibiotics by facilitating the production of capsular polysaccharides (Gallego, 2016). Biofilms can be detected using the polystyrene blue assay, which relies on crystal violet staining. This method is in use but can be less sensitive and is generally more labor-intensive.

Statistical analysis by Yang et al. revealed that the *ompA* and *bap* genes play a significant role in *A. baumannii* biofilm formation and antimicrobial resistance (Yang et al., 2019). The study found that the *ompA* gene was prevalent in 68.8% of the *A. baumannii* isolates and plays a crucial role in biofilm formation and antimicrobial resistance. *ompA* aids in surface adhesion and contributes to biofilm initiation, which aligns with its role in enhancing bacterial survival and evading immune responses. In contrast, the *bap* gene, found in 79.2% of the isolates, was similarly linked to biofilm formation and antimicrobial resistance. It is essential for biofilm maturation, promoting bacterial aggregation and stabilizing the biofilm structure, which in turn contributes to reduced antibiotic penetration and increased bacterial persistence (Yang et al., 2019).

In colistin resistance, detecting resistance in *A. baumannii* poses significant challenges due to the complexity of resistance mechanisms, particularly the presence of *mcr* genes and modifications in the pmrCAB operon. Phenotypic methods like broth microdilution (BMD) are considered the gold standard. Still, they are time-consuming, while molecular techniques such as PCR offer precise detection of mcr genes but require specialized resources (Novović and Jovčić, 2023). The most thorough detection strategy is a hybrid approach that combines the two techniques. BMD and PCR are advised for accurate detection in high-resource environments, whereas disk elution and other more straightforward phenotypic tests are more useful in low-resource environments (Arroyo et al., 2005). Molecular epidemiology relies on PCR for tracking resistance patterns and outbreaks. Additionally, mechanisms such as the loss of LPS production contribute to colistin resistance, highlighting the need for diverse diagnostic approaches (Moffatt et al., 2010).

#### 7.1.2 Conventional molecular methods

Currently, the definitive diagnosis and identification of *A. baumannii* are primarily accomplished through molecular methods, particularly PCR, which is used extensively for confirmation of *A. baumannii* and the detection of resistance genes to carbapenems (Turton et al., 2006). This method, also known as random amplified polymorphic DNA (RAPD), can be employed in investigations to trace local spread during epidemics (Pourcel et al., 2011). PCR is widely used due to its sensitivity and specificity, but it can be time-consuming and requires laboratory infrastructure for reliable results.

In addition to traditional PCR, several other techniques, such as PCR replicon typing, multiplex PCR, and multiplex RT-PCR, offer accurate and efficient screening methods for various bacterial strains and genetic targets. Polymerase chain reaction-electrospray ionization mass spectrometry (PCR-ESI/MS) provides broad-based capabilities for detecting samples, specifically identifying silent mutations and genetic rearrangements of target genes (Wolk et al., 2012). While promising, PCR-ESI/MS is still under development and not widely used in clinical settings, and the majority are currently available only for research use. It requires specialized equipment and is relatively expensive and time-consuming compared to traditional PCR (Wolk et al., 2012).

Moreover, repetitive sequence-based PCR (rep-PCR) and multiple locus variable number of tandem repeat analysis (MLVA) enable the detection of repetitive sequences in the bacterial genome and tandem repeats, respectively (Rafei et al., 2014). While rep-PCR is mainly used for genotyping rather than routine clinical detection of *A. baumannii*, MLVA has been applied in clinical settings for outbreak surveillance and epidemiological studies due to its high discriminatory power. However, MLVA's use in routine diagnostics is limited by the need for specialized analysis tools and potential variability in repeat numbers, which may impact reproducibility (Anwer, 2024). PFGE is a method for studying the clonality of environmental and clinical isolates (Goodman, 1988). Widely regarded as the gold standard in epidemiological investigations, PFGE is instrumental in understanding the spread of pathogens (Zarrilli et al., 2013). However, it is time-consuming and requires specialized equipment, making it less practical for routine use in clinical diagnostics.

#### 7.1.3 Emerging and novel tools

Various typing methods are employed in large-scale epidemiological studies, including ribotyping and amplified fragment length polymorphism (AFLP). Sequence-based approaches, such as single-locus typing (e.g., adeB, rpoB, blaOXA-51-like, and recA) or multilocus sequencing (e.g., 3-LST, trilocus sequencing), are also utilized to characterize *A. baumannii* isolates (Rafei et al., 2014). These methods offer high precision but are more complex and require advanced infrastructure, limiting their widespread application in clinical diagnostics (Anwer, 2024).

Furthermore, loop-mediated isothermal amplification (LAMP) has emerged as a rapid and highly sensitive tool for detecting carbapenemase genes associated with *A. baumannii* resistance, notably ISAba1-blaOXA-51-like. LAMP offers advantages such as cost-effectiveness and fast processing, requiring only 21 min for the entire procedure (Mu et al., 2016). This method enables amplification without the prior purification of the pathogen's DNA and allows for sensitive detection of genes, such as recA and the carbapenemase OXA-23. LAMP is practical, inexpensive, and faster than many PCR-based methods, making it a strong candidate for use in resource-limited settings. Additionally, multiplex LAMP can be employed for the simultaneous detection of multiple genes, further enhancing its practicality for rapid, comprehensive diagnostics (Yang et al., 2018).

The lab-on-a-disk (LOAD) specimen method is a sample-to-answer diagnostic approach for bacterial infections. It offers simplicity and automation across all its steps, including DNA extraction and purification from bacteria, targeted genetic marker amplification via LAMP reaction, and signal detection. The LOAD platform demonstrates high specificity and rapidity, providing results within 2 h. This makes it particularly valuable for critically ill patients at risk of shock or sepsis, facilitating early administration of appropriate treatment. Notably, its microfluidic setup enables operation independent of fluid composition (Loo et al., 2017). The rapid turnaround time of LOAD makes it a promising method for point-of-care settings. However, it is still under development and may not be widely available in clinical practice.

Fatmawati et al. emphasized the efficacy of a multidisciplinary approach, combining various tests such as antibiograms, genetic profiling of *OXA-23* genes, and random amplified polymorphism DNA polymerase chain reaction (RAPD-PCR), in surveillance systems. Particularly beneficial in hospitals with limited microbiology laboratories, this cost-effective strategy enables early assessment of MDR *A. baumannii*. Furthermore, it holds promise for extending detection to other MDR bacteria (Fatmawati et al., 2023).

### 7.2 Challenges in accurate diagnosis

Several challenges hinder the accurate diagnosis of *A. baumannii* infections, foremost among them being the incomplete understanding of its virulence mechanisms. Furthermore, the intricate interplay between virulence factors and antimicrobial resistance complicates diagnostic efforts (Wong et al., 2017).

For instance, drug-resistant strains pose a significant challenge in lateral flow immunoassays (LFIA), a technique reliant on antibodies targeting specific bacterial antigens. LFIA offers practicality and rapidity in detection (Wang et al., 2022, 2023b). In the case of *A. baumannii*, monoclonal antibodies targeting genes such as OXA-28 and OXA-40 can aid in assessing the bacterium's carbapenem resistance clinically (Mertins et al., 2023). However, drug-resistant strains may exhibit antigenic variation or altered expression of resistance markers, reducing monoclonal antibodies (mAbs) binding efficiency and leading to false negatives in LFIA tests. Additionally, some carbapenemase-producing strains express diverse OXA variants, which may not be recognized by the antibodies used in standard LFIA assays. These challenges are particularly relevant in clinical settings, where timely and accurate detection of resistant strains is crucial for appropriate treatment. Implementation of an LFIA test in the clinical setting has to take into account the local epidemiological context in terms of the prevalence of resistance mechanisms (Boutal et al., 2022).

Furthermore, traditional DNA sequencing methods are time-consuming, costly, and labor-intensive, particularly when analyzing different strains. An innovative approach called PCR-restriction fragment length polymorphism (PCR-RFLP) has emerged to address this. Mismatched PCR-RFLP assays, for instance, have been developed to detect specific genes associated with *A. baumannii*'s resistance to fluoroquinolones, offering rapid and accurate differentiation from other *Acinetobacter* strains (Kakuta et al., 2020).

Regarding PCR-ESI/MS, as mentioned earlier, several potential challenges exist, including the risk of contamination and limitations in identifying mixed infections due to the need to analyze multiple factors. Also, detecting colony-forming units as low as 1–10/mL may pose a limitation. Moreover, PCR-ESI/MS tends to be costlier than existing molecular diagnostic tools (Wolk et al., 2012).

Furthermore, specific molecular techniques for *A. baumannii* detection, such as PCR, DNA sequencing, and MALDI-TOF-MS, have their own limitations, particularly in resource-limited regions. These include high costs and the requirement for highly trained professionals (Wang et al., 2023a).

### 7.3 Novel diagnostic tools and technologies

Traditionally, the DNA–DNA hybridization assay was utilized, but it has since been supplanted by WGS methods and single-nucleotide polymorphism (SNP) determination. Currently, Albert *et al*. have demonstrated that a multiplex gyrB PCR assay can be employed for easy *A. baumannii* identification (Albert et al., 2022). While PCR is highly sensitive and specific, it requires specialized equipment and trained personnel, making it less cost-effective in resource-limited settings compared to newer technologies. Moreover, NALFA, short for nucleic acid amplification lateral flow assay, offers minimal development costs and easy production, making it highly cost-effective. It boasts a short immunoassay reaction time of <20 min and can detect various drug-resistant bacteria on the same paper without amplifying specific antibodies. NALFA is advantageous in its simplicity, lower cost, and rapid turnaround, but it may not match the sensitivity of PCR or mass spectrometry methods (Boutal et al., 2022).

On the contrary, the LAMP technique, the most developed isothermal amplification method, is cost-effective and simple, but requires specialized personnel for result interpretation and may take longer than an hour. Hence, a novel technology combining both methods has been innovated, specifically for detecting *A. baumannii* and CRAB. This method offers a quick and particular diagnostic test that can be completed in <43 min, combining the low cost and ease of NALFA with the sensitivity of LAMP. It also eliminates the need for specialized personnel, thus enhancing its practicality in diverse healthcare settings (Wang et al., 2023a).

In addition, MALDI-TOF-MS is another method capable of identifying and confirming bacterial species, making it suitable for detecting *A. baumannii* (Owusu et al., 2023). With a sensitivity of 100% for *A. baumannii* detection, it exhibits a specificity ranging from 91.7 to 99% (Vijayakumar et al., 2019). While MALDI-TOF-MS provides rapid results (typically within minutes) and offers high specificity and sensitivity, it requires a significant initial investment in equipment and regular maintenance, which might not be feasible for low-resource hospitals. As the technology continues to develop and costs decrease, it is expected that these methods will become more widespread and accessible (Jacobs et al., 2021).

Furthermore, the Vitek2 Compact system, utilizing ID-GNs cards, has demonstrated a 99% accuracy rate in diagnosing *A. baumannii* compared to traditional methods (Manfi Ahmed et al., 2022). This system is efficient and offers rapid results, but its higher cost, limited ability to distinguish between species within the *A. baumannii* complex, and the need for trained personnel limit its applicability (Lee et al., 2014).

Finally, in artificial intelligence (AI), a machine learning system has been developed and proven effective in the early detection of *A. baumannii* infection and/or colonization. This system utilizes data from laboratory tests and time series chest X-rays to aid in administering individualized treatments and achieving better outcomes by estimating risks. While promising, this technique requires further validation through additional prospective large-scale trials and studies, as suggested by Zeng et al. (2024). The main advantage of AI systems lies in their potential for personalized treatment, although current limitations include the need for extensive data and further refinement in accuracy.

## 8 Treatment strategies for *A. Baumannii* infections

Despite advancements in antimicrobial therapy, *A. baumannii* remains an essential but challenging-to-treat pathogen, owing to its tendency to develop resistance and to cause severe clinical complications. The antibiotic options for treating *A. baumannii* are remarkably restricted due to high rates of resistance. Nonetheless, different antibiotic treatment modalities, including empirical and targeted therapies and alternative non-antibiotic treatments, are opted for the optimal management of *A. baumannii* infection (Table 4).

**Table 4:** Summary of treatment strategies for *Acinetobacter baumannii* infections.

**Treatment strategies**		**Examples**	**References**
Antibiotic therapies	First-line antibiotics	Carbapenems (meropenem, imipenem)	Song et al., 2019; Liebchen et al., 2020; Kim A. et al., 2009; Papp-Wallace et al., 2011; Henry, 2019; Garnacho-Montero et al., 2015
		Fluoroquinolones (ciprofloxacin, levofloxacin)	Abdul-Mutakabbir et al., 2021
		Aminoglycosides (amikacin, gentamicin)	Vidal et al., 2007; Thy et al., 2023
	Second-line antibiotics	Polymyxins (polymyxin B, colistin)	Tamma et al., 2020; Batirel et al., 2014; Garnacho-Montero et al., 2003; Intravenous, 1999
		Tetracycline derivatives (tigecycline, minocycline)	Greig and Scott, 2016; Ritchie and Garavaglia-Wilson, 2014; Zhanel et al., 2006; Ni et al., 2016
	Combination therapy	β-Lactam/β-lactamase inhibitors (ampicillin/sulbactam, piperacillin-tazobactam)	Kuo et al., 2007
		Polymyxin + tetracycline derivative or sulbactam	Song et al., 2007; Kempf et al., 2012b
		Newer combinations: durlobactam/sulbactam	Watkins and Bonomo, 2023; Karruli et al., 2023
Non-antibiotic therapies	Phage therapy	Bacteriophage cocktails	Aleshkin et al., 2016; Schooley et al., 2017; Rao et al., 2022
	Immunization-based therapy		
	Monoclonal antibody-mediated therapy	Monoclonal antibodies (e.g., anti-OmpA, mAb C8, anti-*A. baumannii* OMV antibodies)	Luo et al., 2012; Singh et al., 2016; Huang W. et al., 2019; Nielsen et al., 2017
	AMPs	SMAP-29, TP4, mastoparan	Vila-Farres et al., 2012; Jung et al., 2021; Alsaab et al., 2023; Jaśkiewicz et al., 2019; Ji et al., 2023
	aPDT	Photosensitizers (e.g., riboflavin, chlorophyllin, methylene blue, aloe emodin)	Buchovec et al., 2022; Marcolan De Mello et al., 2019; Li et al., 2020

*A. baumannii, Acinetobacter baumannii*; AMPs, antimicrobial peptides; aPDT, antimicrobial photodynamic therapy; OMV, outer membrane vesicle; OmpA, outer membrane protein A.

### 8.1 Antibiotic therapies

In the absence of definitive microbiological pathogen identification and susceptibility testing, the initial antimicrobial regimen involves starting with empirical antimicrobial therapy (EAT). The latter aims to cover potential pathogens based on the patient's clinical history, risk factors, and local epidemiology, thereby halting further complications (Boyd and Nailor, 2011; Luo et al., 2023). Empirical therapy for *A. baumannii* infections necessitates broad-spectrum antibiotics that are active against Gram-negative bacteria. EAT against *A. baumannii* includes first-line and second-line antibiotics.

First-line antibiotics are prescribed for infections caused by relatively antimicrobial-susceptible *A. baumannii* isolates. β-Lactam antibiotics, including carbapenems and β-lactam/β-lactamase inhibitor combinations, are often adopted as first-line treatment. In ICU settings with lower prevalence of CRAB, carbapenems, such as meropenem and imipenem, are usually considered the drugs of choice due to their broad-spectrum potency, safety, and tolerability profile compared to other antibiotics within the same family (Song et al., 2019; Liebchen et al., 2020; Kim A. et al., 2009; Papp-Wallace et al., 2011; Henry, 2019; Garnacho-Montero et al., 2015). Additional empirical strategies include utilizing sulbactam, such as in ampicillin/sulbactam combination modality, which relies on the irreversible intrinsic activity of sulbactam against *A. baumannii* (Smolyakov et al., 2003). Other modalities may involve the use of carbapenems in combination with a β-lactam and a β-lactamase inhibitor, such as piperacillin-tazobactam, to enhance gram-negative coverage and prevent resistance (Kuo et al., 2007). For mild infections, fluoroquinolones, such as ciprofloxacin and levofloxacin, and third-generation cephalosporins may also be considered based on local susceptibility data, despite higher resistance rates (Abdul-Mutakabbir et al., 2021). Aminoglycosides, including amikacin and gentamicin, may sometimes be used in combination with other antibiotics (Vidal et al., 2007; Thy et al., 2023).

Second-line agents are employed in the setting of a high suspicion of CRAB or resistance to the other first-line agents, second-line agents are employed. These agents mainly include polymyxins and tetracycline derivatives, with sulbactam remaining an efficient option (Tamma et al., 2020). Polymyxins, such as polymyxin B and polymyxin E, are reserved for serious MDR infections due to their nephrotoxicity and neurotoxicity profile (Tamma et al., 2020; Batirel et al., 2014; Garnacho-Montero et al., 2003; Intravenous, 1999). Tetracycline derivatives, such as tigecycline and minocycline, are used for their broad-spectrum activity against resistant strains, although tigecycline's low serum concentration limits its use in bloodstream infections (Greig and Scott, 2016; Ritchie and Garavaglia-Wilson, 2014; Zhanel et al., 2006; Ni et al., 2016). Polymyxin-based combination therapy is often recommended for CRAB cases to enhance efficacy and prevent further resistance. The rationale for combination therapy lies in the synergistic or additive effects between agents, enabling for more effective eradication of the infecting pathogen. However, selecting appropriate combinations requires careful consideration of antimicrobial susceptibilities, potential drug interactions, and the risk of toxicity. The common combination regimens include polymyxin with a tetracycline derivative or sulbactam (Kengkla et al., 2018). For instance, colistin or polymyxin B combined with tigecycline or minocycline, or with sulbactam, showed effective selections *in vitro* (Song et al., 2007; Kempf et al., 2012b). Newer sulbactam combinations, such as durlobactam/sulbactam, have emerged as a newer and a step forward treatment for CRAB infections (Watkins and Bonomo, 2023; Karruli et al., 2023).

Despite the efficacy of empirical therapy, it is essential to recognize that over-reliance on these regimens can increase the risk of developing resistance and lead to suboptimal outcomes (Luo et al., 2023). Thus, once the diagnostic testing identifies the causative pathogen, switching from broad-spectrum empirical antibiotics to narrower-spectrum targeted agents is essential to ensure satisfactory outcomes and mitigate the risk of further resistance. Employing targeted antibiotic therapy effectively minimizes side effects and damages to the patient's microbiota and reduces the selective pressure for antimicrobial resistance. By tailoring the treatment to the specific pathogen and its resistance profile, targeted and personalized therapy not only enhances the efficacy of the treatment but also minimizes antibiotic abuse.

Epidemiological data highlight significant geographical variations in MDR/XDR *A. baumannii* prevalence, directly influencing the appropriate antimicrobial treatment selection, especially for those used as empirical therapies. A recent meta-analysis underscores these geographical disparities, revealing notably high carbapenem resistance in Asia, with an incidence of 76.2%, while America reports the lowest incidence at 69.4% (Beig et al., 2024). A more detailed study from the Asia–Pacific region, conducted from 2012 to 2019, revealed that carbapenem resistance rates were as low as 2.8% in Japan and 6.5% in Australia, but reached as high as 88% in South Korea and 87.2% in India. Among the carbapenem-resistant isolates, 96.6% harbored at least one carbapenemase gene, with the *blaOXA-23* gene being the most prevalent. The study also reported significant resistance to other antibiotics, including amikacin, ampicillin/sulbactam, and cefepime, highlighting the ongoing challenge of managing *A. baumannii* infections in regions with high resistance rates (Lee et al., 2023). This geographical variation underscores the importance of understanding local resistance patterns and the limited treatment options.

To guide evidence-based clinical decision-making, several authoritative organizations, including the Infectious Diseases Society of America (IDSA) and the European Society of Clinical Microbiology and Infectious Diseases (ESCMID), provide treatment recommendations for *A. baumannii* infections. For CRAB infections, IDSA recommends combining sulbactam-durlobactam with either meropenem or imipenem-cilastatin as the preferred regimen. High-dose ampicillin-sulbactam, in combination with at least one additional agent, has been downgraded from a preferred to an alternative option if sulbactam-durlobactam is unavailable. The suggested dosing for high-dose ampicillin-sulbactam is 27 grams daily (18 g of ampicillin and 9 g of sulbactam; Tamma et al., 2024). In contrast, ESCMID recommends polymyxin-meropenem combination therapy (high certainty of evidence) and polymyxin-rifampin combination therapy (moderate certainty of evidence) for all patients with CRAB infections. For patients with severe or high-risk CRAB infections, ESCMID suggests combination therapy using two *in vitro* active antibiotics from available options (polymyxin, aminoglycoside, tigecycline, or sulbactam combinations). However, this recommendation is conditional with very low certainty of evidence. For patients with CRAB infections and a meropenem minimum inhibitory concentration (MIC) <8 mg/L, carbapenem combination therapy with high-dose extended-infusion carbapenem dosing is considered good clinical practice (Paul et al., 2022). Consulting local guidelines ensures that treatment strategies align with the regional resistance patterns and available therapeutic options.

### 8.2 Alternative non-antibiotic therapies

To counteract the rising antibiotic resistance and the challenges to discovering and synthesizing new antibiotics, alternative treatment strategies are being explored for managing the emergence and dissemination of MDR *A. baumannii*. These mainly include non-antibiotic therapies such as bacteriophage therapy, immunization-based therapy, monoclonal antibodies, and adjunctive therapies such as antimicrobial peptides and antimicrobial photodynamic therapy.

#### 8.2.1 Phage therapy

Bacteriophages are natural bacterial killers used in practical applications since the early 20th century. In the post-antibiotic era, phagebiotics have received special attention as an alternative treatment option to fight bacterial infections (Wei et al., 2020; Gordillo Altamirano and Barr, 2019). The main advantage of phage therapy over traditional antibiotic treatments lies in the high specificity of phages to their targets, effectively killing pathogenic bacteria without affecting the normal microflora (Rai and Kumar, 2022). Aleshkin et al. conducted a small-scale clinical trial to evaluate the therapeutic efficacy of a phage cocktail in an ICU emergency caused by multiresistant bacterial strains, including *A. baumannii*. After 3 days of administering phage therapy, the infectious contamination level in 14 patients dropped from 79 to 21%, indicating clinical improvement (Aleshkin et al., 2016). Additionally, an 88-year-old patient diagnosed with CRAB-caused HAP was treated with personalized phage lysis preparation in combination with tigecycline and polymyxin E. The treatment was well tolerated and resulted in the clearance of the pathogen and improvement in the patient's lung function (Clemmer et al., 2011). Another study showed that administering *A. baumannii* phage cocktails to a patient with necrotizing pancreatitis, diabetes mellitus, and MDR *A. baumannii* infection gradually improved overall clinical symptoms (Schooley et al., 2017). Indeed, compared with single phage administration, phage–antibiotic combination therapy is a promising treatment option. In a critically ill patient with MDR *A. baumannii* pneumonia, intravenous phage treatment co-administered with antibiotics significantly improved the patient's condition compared to single administrations (Rao et al., 2022).

While phage therapy is gaining prominence as an antibiotic alternative, its clinical implementation faces several challenges. While it is advantageous in reducing off-target effects, the high specificity of bacteriophages necessitates individualized treatment plans that may not be feasible for widespread clinical application. Bacterial resistance to phages and the potential for immune system recognition and neutralization remain significant concerns. Large-scale clinical trials are required to establish standardized protocols, determine optimal delivery methods, and assess long-term safety and efficacy (Pires et al., 2020; Ogungbe et al., 2024).

#### 8.2.2 Immunization-based therapy

Vaccine development targeting antibiotic resistance-associated proteins and virulence factors has gained interest as a proactive approach to preventing infections rather than treating them after occurrence (Micoli et al., 2021). Vaccine candidates against *A. baumannii* have demonstrated promising preclinical efficacy. For instance, vaccination of diabetic mice with recombinant OmpA (rOmpA) markedly improved survival, reduced tissue bacterial burden, and induced high titers of anti-OmpA, which are correlated with better survival in mice (Luo et al., 2012). Another study conducted an *in silico* analysis of the *A. baumannii* ATCC 19606 proteome, predicting that FilF, a conserved OMP, could be a potential vaccine candidate. The study demonstrated that FilF provided immunoprotective efficacy in mice, indicated by increased antibody titers, reduced inflammation, and decreased severity of infection and bacterial burden (Singh et al., 2016). In combination therapy, Huang *et al*. utilized a mouse sepsis model to examine the effect of delivering anti-*A. baumannii* OMV antibodies through both active and passive immunization strategies. The results indicated significant improvements in susceptibility to quinolone antibiotics, higher survival rates, and reduced bacterial loads in the organs (Huang W. et al., 2019). In another pneumonia model, the combination of serum anti-*A. baumannii* OMV and levofloxacin enhanced antibiotic sensitivity and significantly reduced bacterial loads in the lungs and spleen compared to single treatments (Huang W. et al., 2019).

Despite promising preclinical results, developing an effective *A. baumannii* vaccine faces several challenges. The genetic diversity of *A. baumannii* strains complicates the identification of universally protective antigens. Moreover, immunization strategies must balance efficacy with potential adverse effects, particularly in immunocompromised patients. The design and execution of clinical trials for such vaccines are further complicated by the need to select appropriate diseases and target populations, particularly without established immune markers. Further research is essential to optimize antigen selection, evaluate long-term immune responses, and conduct comprehensive clinical trials to assess the effectiveness (Brazzoli et al., 2023; Rumata et al., 2023).

#### 8.2.3 Monoclonal antibody-mediated therapy

Beyond vaccination, mAbs are gaining significant momentum as a viable therapeutic option for infections caused by antibiotic-resistant bacteria. Nielsen et al. reported that a mAb, C8, raised against *A. baumannii* capsule showed a protective effect in bloodstream and lung models of *A. baumannii* infection, including an XDR clinical isolate. This mAb enhanced bacterial clearance, prevented the progression to septic shock, and worked synergistically when combined with colistin (Nielsen et al., 2017). Building on this, recent advances have led to the development of mAb 65, which offers broader strain coverage than C8 and significantly improves survival rates in murine models of bacteremic sepsis and aspiration pneumonia (Nielsen et al., 2017). Notably, mAb 65 enhances bacterial clearance, modulates inflammatory responses, and exhibits synergy with colistin, improving overall treatment outcomes (Nielsen et al., 2017). In another study, utilizing a human immune repertoire mouse model, researchers identified 297 antibodies targeting clinically relevant *A. baumannii* strains and analyzed 26 for functional potential. Among these, mAb1416, which specifically targets the KL49 capsular polysaccharide, demonstrated prophylactic efficacy *in vivo* against CRAB lineages associated with neonatal sepsis mortality (Baker et al., 2024). Additionally, *Yeganeh* et al. developed novel mAbs against OmpA of *A. baumannii*, with 1G1-E7 showing strong bacterial reactivity and significantly enhancing opsonophagocytic killing of a clinical isolate. These findings highlight OmpA as a promising target for antibody-mediated therapies against *A. baumannii* infections (Yeganeh et al., 2021).

Monoclonal antibody therapy offers a targeted approach to combating MDR *A. baumannii* infections, but high production costs and complex manufacturing processes may limit accessibility. Variations in bacterial surface antigens can reduce efficacy across different strains, while potential immunogenicity and the need for repeated dosing present additional challenges. Addressing these limitations requires advances in biotechnology, optimized delivery strategies, and rigorous clinical research to ensure safety, efficacy, and cost-effectiveness (Luo et al., 2025).

#### 8.2.4 Antimicrobial peptides

Antimicrobial peptides (AMPs) emerge as promising new antimicrobial agents with significant potential for treating *A. baumannii* infections. These peptides, also known as host defense peptides, are naturally produced by nearly all living organisms as part of their innate immune system to combat pathogens (Fan et al., 2016; Bucataru and Ciobanasu, 2024). Numerous AMPs are currently under investigation to determine their therapeutic efficacy against *A. baumannii* strains, and various *in vitro* and *in vivo* studies have demonstrated that several AMPs exhibit antimicrobial activity against MDR *A. baumannii* (Vila-Farres et al., 2012; Jung et al., 2021; Alsaab et al., 2023; Jaśkiewicz et al., 2019; Ji et al., 2023). For instance, an *in vivo* study using a mouse model identified two AMPs, SMAP-29 and TP4, as having prophylactic effects that prevented mortality in mice with *A. baumannii*-induced pneumonia. Additionally, two derivatives of TP4, dN4 and dC4, showed therapeutic efficacy in pneumonia mouse models when administered peritoneally or intravenously. These derivatives also effectively inhibited and/or eradicated *A. baumannii* biofilms at higher doses (Jung et al., 2021). Moreover, research by Vila-Farres *et al*. examined the activity of 15 peptides against both colistin-susceptible and colistin-resistant *A. baumannii* strains *in vitro*. Among the AMPs tested, mastoparan displayed notable bactericidal activity at a concentration 8 times the minimum inhibitory concentration (MIC x8) against both strains, indicating its potential as an alternative treatment for colistin-resistant *A. baumannii* infections (Vila-Farres et al., 2012). Interestingly, when mastoparan was co-administered with colistin in another study, a 100% synergistic effect was observed against 24 XDR *A. baumannii* strains (Kim et al., 2023). Despite their promising antimicrobial potential, AMPs face several challenges that hinder their clinical translation. High production costs, susceptibility to enzymatic degradation, and potential toxicity at therapeutic concentrations limit their widespread use. Additionally, the complex and lengthy drug development process, coupled with the incomplete understanding of their mechanism of action, poses further barriers to their optimization and regulatory approval. As a result, only a limited number of AMPs have progressed through clinical trials, with even fewer receiving FDA approval for clinical use (Bucataru and Ciobanasu, 2024; Mishra et al., 2025).

#### 8.2.5 Antimicrobial photodynamic therapy

Antimicrobial photodynamic therapy (aPDT) offers a promising alternative and complementary approach to battle MDR *A. baumannii* (Bustamante and Palavecino, 2023). This technique combines a photosensitizer, light, and oxygen to produce reactive oxygen species (ROS), which exert bactericidal or bacteriostatic effects by inducing photooxidative stress on bacterial structures. This method is beneficial for localized infections and minimizes damage to surrounding healthy tissues (da Fonseca et al., 2021; Piksa et al., 2023; Rajesh et al., 2011). One study evaluated the efficacy of aPDT based on natural photosensitizers, riboflavin- and chlorophyllin, using near-ultraviolet or blue light on *A. baumannii* biofilms. The study found that the antibacterial effect of riboflavin-based aPDT depends on photoactivated riboflavin's ability to generate intracellular ROS. Additionally, chlorophyllin-based aPDT's effectiveness was linked to the sensitivity of *A. baumannii* biofilms to light exposure. The results significantly impacted bacterial viability, structural stability, and ROS generation (Buchovec et al., 2022). Another study evaluated the effect of aPDT against carbapenem-susceptible and CRAB strains, using methylene blue and a low-level laser. The results showed a decline in *A. baumannii* cells across all isolates tested, with reductions ranging from 63 to 88% for susceptible isolates and 26–97% for resistant ones (Marcolan De Mello et al., 2019). Further research evaluated the photodynamic inactivation (PDI) efficacy mediated by aloe emodin (AE), a natural compound isolated from *Aloe vera* and *Rheum palmatum*, against MDR *A. baumannii* isolates *in vitro*. AE exhibited no significant dark toxicity and effectively inactivated clinical isolates in a concentration -and light-dose-dependent manner. The study revealed that AE-mediated aPDT caused genomic DNA damage and significant bacterial membrane disruption, as evidenced by the LIVE/DEAD ratio. These findings suggest that AE is a promising photosensitizer for aPDT treatment of infections caused by MDR *A. baumannii* (Li et al., 2020).

Despite its potential, aPDT faces several limitations, including limited penetration depth of light, potential damage to host tissues, and the need for precise dosing to avoid cytotoxic effects. The efficacy of aPDT is also influenced by bacterial resistance mechanisms and variations in light absorption properties among different strains. More extensive clinical research is required to determine optimal photosensitizers, refine light delivery techniques, and assess long-term safety for use in human infections (Bustamante and Palavecino, 2023).

Though these alternative treatments hold great promise, further research is needed to establish their efficacy, safety, and clinical applicability in the management of *A. baumannii* infections. Rigorous clinical trials and studies are essential to understand these therapies' full potential and limitations, ensuring they can be effectively integrated into clinical practice to improve patient outcomes and combat the growing threat of antibiotic resistance.

## 9 Infection control and prevention

### 9.1 Strategies for preventing transmission in healthcare settings

#### 9.1.1 Antimicrobial stewardship

Antimicrobial stewardship is a comprehensive program that entails antibiotic restriction policies aimed at optimizing antimicrobial use, reducing the incidence of infection, improving patients' clinical outcomes, ensuring minimum unintended consequences, and saving on unnecessary healthcare costs (Garnacho-Montero et al., 2015; WHO, 2019). It has been suggested as a way to manage *A. baumannii*-related outbreaks, as the proportion of patients in the ICU who are infected has decreased (Cheon et al., 2016). The intention is to stop circulating strains of *A. baumannii* from developing resistance in the future (Bianco et al., 2016). For a better adoption of its maximum interventions, basic continuous education of healthcare workers (HCWs) is necessary to improve the prescribing behaviors (Orok et al., 2025). Prospective or retrospective auditing, which provides feedback about the prescribed antibiotic treatment and de-escalation, which shifts away from broad-spectrum antibiotics to more targeted options, are key feedback interventions in this program that minimize the risks associated with inappropriate antibiotic use (WHO, 2019; Akpan et al., 2016). As part of this stewardship, a restricted list of authorized drugs for colistin, tigecycline, carbapenems, and fluoroquinolones is enforced to reduce antibiotic use and improve antibiotic prescribing practices (Meschiari et al., 2021).

#### 9.1.2 Education

Adequate knowledge of infection prevention and control is crucial for preventing HAIs, which affect up to 37% of ICU patients. Despite knowledge, compliance with hand hygiene and personal protective equipment (PPE) use varied widely, so regular education and training should be implemented (Alhumaid et al., 2021). Training sessions were held for hospital staff to raise their compliance with hand hygiene protocols and to enhance their knowledge about why it is crucial to prevent the spread of *A. baumannii* (Cheon et al., 2016). The importance of following standard precautions was highlighted through educational sessions and reminders, which were integrated into a training program that also included a sociological component (Tanguy et al., 2017). To ensure that this measure is implemented, screensavers with hand hygiene guidelines downloaded from the WHO website were installed on all computers in the hospital (Cheon et al., 2016).

#### 9.1.3 Hand hygiene and contact precautions

It is imperative for healthcare providers to rigorously adhere to standard and contact precautions that include hand hygiene before and after glove removal, as well as disposable gown and glove removal before exiting the room, to effectively suppress the transmission of infections (Tanguy et al., 2017; WHO, 2022). To facilitate proper hand hygiene in the ICUs, alcohol-based hand rub was made readily accessible at the bedside for all patients. Additionally, a non-alcoholic hand sanitizer was provided to any HCW who reported allergies to alcohol. Patients who tested positive for *A. baumannii* were included in a cohort in a designated area within each unit, and contact precautions were enforced (Cheon et al., 2016).

Moreover, in ICUs lacking sufficient isolation rooms or where the proximity of the beds presents a concern, patients were separated using transparent plastic curtains (Cheon et al., 2016). It aims to prevent cross-transmission between these patients along with the use of specialized personal protective equipment such as long-sleeved gowns, FFP3 masks, and face visors (Warde et al., 2019). However, antimicrobial curtains impregnated with silver or treated with quaternary ammonium chlorides were able to reduce the contamination by a significant margin compared to standard hospital ones (Luk et al., 2019). As an alternative to curtains, thick red lines painted on the floor can function as “virtual walls,” marking the boundaries of each patient's personal space, where passing through it in either direction means the performance of hand hygiene. This was associated with a decrease in the risk of acquiring resistant pathogens (Ben-Chetrit et al., 2018).

### 9.2 Environmental disinfection

#### 9.2.1 Daily and post-discharge cleaning

Routine cleaning of rooms and surfaces was conducted daily, and after the discharge of every patient. Environmental cleaning using a chlorine-based disinfectants (10% sodium hypochlorite) was done at least twice per day in addition to hydrogen peroxide wipes for cleaning the medical devices (Meschiari et al., 2021; Tanguy et al., 2017; Warde et al., 2019). For sensitive electronic equipment, quaternary ammonium-containing wipes were employed (Ben-Chetrit et al., 2018). The concentration of disinfectants was carefully controlled to ensure effective cleaning, and surfaces are left to dry completely before being used again (Chmielarczyk et al., 2012).

#### 9.2.2 Patient relocation and skin disinfection

Infected patients were temporarily moved to a transitory unit while their original rooms were disinfected. In this unit, the patient's skin was disinfected with 2% leave-on chlorhexidine disposable cloths or decolonized with octenidine-containing products, which have been shown to reduce the number of positive cases. Afterward, the patient was transferred back to the cleaned unit (Meschiari et al., 2021).

#### 9.2.3 Terminal disinfection

When conventional cleaning approaches proved insufficient in preventing the spread of *A. baumannii*, a cutting-edge technique was employed. It utilized vaporized hydrogen peroxide (VHP) for terminal disinfection. VHP is a powerful disinfectant that eliminates a wide range of hospital-acquired pathogens while being safe for use, as it ultimately breaks down into harmless oxygen and water (Chmielarczyk et al., 2012). The disinfection was carried out using the Nocospray^®^ (Oxy'Pharm, Champigny-sur-Marne, France) system, with the room sealed and emptied for the process to be effective (Meschiari et al., 2021; Tanguy et al., 2017). Moreover, ultraviolet-C (UV-C) of 222-nm wavelength, which damages the genetic material, was used for high-touch surfaces to avoid the possible deterioration of the material from long-term alcohol wipes. The effectiveness of UV-C light might depend on factors such as exposure time, dose, and UV intensity for each material, which necessitates further research to evaluate its potential applications fully and to assess the optimal exposure conditions (Huang et al., 2024).

#### 9.2.4 Contamination control measures

Hospital rooms gained advantages from the use of portable HEPA filters, as they have been shown to lower airborne *A. baumannii* levels significantly (Barbut et al., 2013). To lessen the environmental contamination, closed-suction systems were also used for patients whose respiratory secretions contained *A. baumannii*. However, new protocols for aseptic methods during open suctioning were implemented for patients not requiring mechanical ventilation (Choi et al., 2010). The results of environmental control measures were reported to ICU head nurses, and subsequent cleaning efforts were focused on areas that yielded positive results for *A. baumannii* and near-patient ones (Cheon et al., 2016). Additionally, the Microflow α microbiological air sampler can be used to analyze the surrounding air, while fluorescent spray and UV torch were used to examine surfaces for the sake of assessing the thoroughness of terminal cleaning (Bianco et al., 2016; Meschiari et al., 2021).

Various techniques are under investigation to reduce *A. baumannii* prevalence in the ICU, although some may be harmful to people or surface materials. Owing to its resistance to the majority of sanitizers and antibiotics, phage φAB2 is recommended as an alternative for environmental decontamination, with close attention to concentration and incubation time (Chen et al., 2013).

### 9.3 Surveillance and outbreak management

The bacteria (culture and sensitivity) were identified using the agar diffusion method, and the VITEK^®^ system (bioMérieux, Marcy-l'Étoile, France), while identification was performed using the MALDI-TOF-MS system (Tanguy et al., 2017; Sousa et al., 2014).

All ICU patients were tested for *A. baumannii* when admitted and continued to have weekly surveillance cultures until they were discharged or had three negative results. Samples collected for patient screening included swabs from the nose, groin, and throat, wound swabs, urine samples, a sputum sample, and potentially a stool sample. If the patient was intubated or had a tracheostomy, tracheal aspirate was obtained. Staff screening involved taking swabs from their nasal passages and skin lesions to monitor potential colonization. This was particularly important in identifying temporary colonization due to their frequent interactions with ventilators and other equipment used in patient care. Furthermore, a study was conducted among staff members to identify any temporary colonization due to their frequent interactions with ventilators and other equipment used in patient care (Cheon et al., 2016; Warde et al., 2019).

Testing was accomplished to evaluate the surroundings of patients who were colonized or infected with *A. baumannii*. Samples from a variety of surfaces, including bed rails, bedside tables, infusion pumps, blood pressure cuffs, supply carts, stethoscopes, notes trolleys, keyboards, shower handles, bathtub walls, floors, suction equipment, pillows, mattresses, sinks, washbasins, water taps, ventilator control panels, and external surfaces of patients' endotracheal tubes on mechanical ventilation, were collected both before and after the rooms were properly cleaned. A pre-moistened, sterile cotton swab can be used to test the environmental and hand cultures. After that, the swab is inoculated onto MacConkey agar plates (Cheon et al., 2016; Barbut et al., 2013; Choi et al., 2010; Alfandari et al., 2014).

An outbreak control team was put together during the outbreak and consisted of infectious disease physicians, a microbiologist, an infection control nurse, and senior nursing staff from the ICU. The first strategy for controlling the outbreak was to halt its transmission through strict contact precautions and intensified infection control measures. The second approach involved minimizing environmental contamination through sterilization and disinfection. Using a closed-suction system was the third strategy (Choi et al., 2010). Furthermore, meetings were conducted with HCWs to offer training periods and to address any potential flaws in the practices (Bianco et al., 2016). Proactive isolation of all new patients until they test negative may be required if standard isolation measures fail to stop the outbreak; if not, the ICU may need to close temporarily (Meschiari et al., 2021; Barbut et al., 2013). Modern means of communication, such as electronic alert systems utilizing GLIMS software (MIPS, a CliniSys Group company, Ghent, Belgium), can be beneficial by notifying whenever a positive sample is confirmed by the bacteriology laboratory, making work easier (Tanguy et al., 2017).

### 9.4 Role of infection control

Implementing comprehensive infection control measures is a critical intervention for effectively managing outbreaks related to *A. baumannii*. These measures included screening patients, separating affected individuals, promoting hand hygiene compliance, putting programs in place to prolong the effectiveness of antibiotics, and improving environmental cleaning and disinfection (Garnacho-Montero et al., 2015; Barbut et al., 2013). Applying these strategies simultaneously led to achieving success in reducing hospital-acquired infection rates and containing outbreaks of *A. baumannii*. Additionally, surveillance and monitoring help identify outbreaks early, enabling prompt intervention to contain the spread of the bacteria.

In summary, preventing and managing *A. baumannii* infections in healthcare settings requires a comprehensive approach, including antimicrobial stewardship, infection control measures, and environmental disinfection. Key strategies include optimizing antibiotic use, promoting hand hygiene and contact precautions, and implementing effective cleaning protocols. Surveillance and outbreak management, regular patient and staff screening, and electronic alert systems are crucial for early detection and intervention. Future research should start helping staff practice creating realistic outbreak scenarios, using virtual reality technology and AI-powered robots for real-time disinfection, to prevent widespread outbreaks.

## 10 Public health implications

### 10.1 Economic burden of *A. Baumannii* infections

*A. baumannii* infections impose a substantial financial burden on healthcare systems worldwide. These infections frequently result in extended hospital stays, larger number of ICU admissions, more need for invasive procedures, and extensive use of broad-spectrum antibiotics, all of which significantly drive up healthcare costs.

A study by Lee et al. on 252 patients with monomicrobial *A. baumannii* infections revealed that the overall 14-day mortality rate in cases of bacteremia was as high as 29.8% (Lee et al., 2012b). More recent studies have confirmed these findings, with *A. baumannii*-related infections continuing to be associated with high mortality rates and economic costs (Alrahmany et al., 2022; Appaneal et al., 2022). The financial burden is particularly true in cases of MDR strains of *A. baumannii*. Compared with non-MDR bacteremia, patients with MDR *A. baumannii* bacteremia experience a significantly higher severity of illness at onset, extended hospitalization (mean duration: 29 days vs. 22.5 days), and increased bacteremia-related mortality (56.2% vs. 4.7%; Zhou et al., 2019).

In addition to direct healthcare expenditures, *A. baumannii* infections also result in substantial indirect costs, such as productivity losses due to prolonged illness, long-term disability, and increased caregiver burden. For example, according to a study by the Public Health Computational and Operations Research (PHICOR) group at the University of Pittsburgh, Pittsburgh, PA, the mean cost to a hospital of each *A. baumannii* case was US$8,246 ± US$4,472 while the costs to the hospital ranged from US$7.4–26.1 million during the period 2006–2007 (Lee B. Y. et al., 2010). The need for expensive last-line antibiotics and heightened infection control measures further adds to the financial strain on healthcare systems (Antunes et al., 2014).

### 10.2 Impact on healthcare systems and patient outcomes: mortality and morbidity impact

The impact of *A. baumannii* infections on the healthcare systems worldwide extends beyond economic costs to profoundly affect patient outcomes and overall quality of care provided. Infections caused by MDR *A. baumannii* often occur in critically ill patients, particularly those in ICUs, causing severe complications, such as VAP, bloodstream infections, and meningitis, as previously discussed (Kanafani et al., 2018; Huang Y. et al., 2019; Jaruratanasirikul et al., 2019; Lynch et al., 2017; Sharma et al., 2019; Pan et al., 2018; Chang et al., 2000; Kelkar et al., 1989; Rodríguez Guardado et al., 2001; Diep et al., 2023). Recent epidemiological data indicate that MDR *A. baumannii* infections have case fatality rates ranging from 29 to 73%, depending on the site of infection and underlying patient conditions, and exceeding 90% in patients with septic shock (Appaneal et al., 2022; Corcione et al., 2024; Russo et al., 2018; Shorr et al., 2014; Tokur et al., 2022).

A significant factor contributing to the poor prognosis of *A. baumannii* infections is its high level of antimicrobial resistance, limiting the treatment options and necessitating last-resort antibiotics such as carbapenems and colistin, which are associated with significant side effects. This situation complicates the management of *A. baumannii* infections and eventually strains healthcare resources and imposing rigorous infection control measures (Peleg et al., 2008). Therefore, it is essential to emphasize the significance of global surveillance and the implementation of effective infection control measures to curb the spread of *A. baumannii* (Falagas and Karveli, 2007). Recent data from global surveillance programs indicate an alarming rise in XDR and PDR *A. baumannii* isolates, complicating treatment efforts even further (Quraini et al., 2023).

The increasing prevalence of drug-resistant *A. baumannii* strains underscores the urgent need for novel therapeutic approaches to overcome the issue of antibiotic resistance, including bacteriophage therapy, antimicrobial peptides, and combination antibiotic regimens, some of which are currently under clinical investigation (Li et al., 2024; Choi and Kim, 2024; Oladipo et al., 2025).

### 10.3 Future directions for research and intervention

Due to its ability to persist in healthcare environments and survive on surfaces for extended periods, *A. baumannii* presents a significant infection control challenge. Stringent measures are required to limit its spread, particularly in hospitals, nursing homes, rehabilitation centers, outpatient clinics, dialysis centers, urgent care centers, hospices, and military medical facilities, which is crucial to prevent the spread of *A. baumannii*. This includes implementing strict hand hygiene practices, which were found to be the most effective intervention (Doan et al., 2016), environmental cleaning, although it is challenging to eradicate *A. baumannii* even after terminal cleaning (Manian et al., 2011; Russotto et al., 2015), and active surveillance for MDR *A. baumannii* colonization by monitoring adherence to infection control guidelines. Nevertheless, infection control still presents a challenge, where even after four rounds of terminal cleaning and disinfection, Manian et al. reported that 26.6% of rooms newly vacated by patients with MDR *A. baumannii* had at least one positive culture site (Manian et al., 2011). International collaboration between researchers, healthcare providers, and public health authorities is essential to address this issue. Data sharing and molecular surveillance programs, such as the *A. baumannii* database hosted by PubMLST.org, facilitate tracking of resistance patterns and outbreak investigations (Bartual et al., 2005; Jolley et al., 2018; Diancourt et al., 2010; Gaiarsa et al., 2019).

## 11 Conclusion

Substantial strides have been attained in our understanding of *A. baumannii*, yet many perplexing mysteries abide. As a successful nosocomial pathogen, *A. baumannii* infection can be hospital-acquired, community-acquired, or even war-acquired. This literature review sheds light on this bacterium's infamy for the association with severe infections, antibiotic resistance, and high mortality and morbidity, underscoring the urgency of early recognition through advancements of diagnostic techniques and effective management strategies. As expounded upon previously, *A. baumannii* can achieve antibiotic resistance through almost all bacterial resistance mechanisms, mainly via enzymatic warfare, reduced membrane permeability, increased efflux mechanisms, target site modification, and genetic mechanisms of resistance. Moreover, a deeper understanding of *A. baumannii*'s virulence factors and pathogenicity promotes future potential targets for therapeutic alternatives and even vaccine development, which is of exceptional importance to control such a pathogen. Particularly noteworthy are the strategies for preventing transmission in healthcare settings, including antibiotic stewardship and surveillance. Ultimately, *A. baumannii* is an affliction for both the economic and healthcare sectors, which makes this understanding essential for hospitals to invest more in controlling the spread of this infection, especially in the ICUs.

In conclusion, *A. baumannii* remains a formidable pathogen, with its ability to acquire AMR posing a significant threat to healthcare and public health systems globally. The evolving resistance mechanisms, particularly to carbapenems and colistin, underscore the urgent need to develop new therapeutic strategies. This review highlights the critical gaps in current treatment options and the importance of exploring novel approaches, such as antibiotic combinations, immunotherapies, and vaccines, which offer promising alternatives to traditional antibiotics.

The findings of this review should inform health policy decisions, particularly in implementing stricter infection control practices and antibiotic stewardship programs in healthcare settings, especially in ICUs, where *A. baumannii* poses the greatest threat. Moreover, as resistance rates continue to rise, investment in research for new drugs and diagnostic tools must be prioritized at the global level.

Given the clinical and global public health significance of *A. baumannii* infections, efforts to combat this pathogen must be intensified. This requires the collaboration of healthcare providers and researchers to drive innovative solutions. The urgency of addressing AMR in *A. baumannii* cannot be overstated. Only through international cooperation and the development of cutting-edge therapies can we hope to effectively manage and ultimately control the spread of this highly resistant pathogen.
